# Hazard potential of Swiss *Ixodes ricinus* ticks: Virome composition and presence of selected bacterial and protozoan pathogens

**DOI:** 10.1371/journal.pone.0290942

**Published:** 2023-11-13

**Authors:** Stefanie Stegmüller, Weihong Qi, Paul R. Torgerson, Cornel Fraefel, Jakub Kubacki

**Affiliations:** 1 Institute of Virology, Vetsuisse Faculty, University of Zurich, Zurich, Switzerland; 2 Functional Genomics Center Zurich, Zurich, Switzerland; 3 Section of Epidemiology, Vetsuisse Faculty, University of Zurich, Zurich, Switzerland; 4 Institute of Virology and Immunology, Mittelhäusern, Switzerland; 5 Department of Infectious Diseases and Pathobiology (DIP), Vetsuisse Faculty, University of Bern, Bern, Switzerland; University of Bologna / Romagna Local Health Authority, ITALY

## Abstract

Ticks play an important role in transmitting many different emerging zoonotic pathogens that pose a significant threat to human and animal health. In Switzerland and abroad, the number of tick-borne diseases, in particular tick-borne encephalitis (TBE), has been increasing over the last few years. Thus, it remains essential to investigate the pathogen spectrum of ticks to rapidly detect emerging pathogens and initiate the necessary measures. To assess the risk of tick-borne diseases in different regions of Switzerland, we collected a total of 10’286 ticks from rural and urban areas in ten cantons in 2021 and 2022. Ticks were pooled according to species, developmental stage, gender, and collection site, and analyzed using next generation sequencing (NGS) and quantitative polymerase chain reaction (qPCR). The metagenomic analysis revealed for the first time the presence of Alongshan virus (ALSV) in Swiss ticks. Interestingly, the pool-prevalence of ALSV was higher than that of tick-borne encephalitis virus (TBEV). Furthermore, several TBEV foci have been identified and pool prevalence of selected non-viral pathogens determined.

## Introduction

Ticks and mosquitos are the primary vectors for transmission of pathogens to humans and animals [1]. The incidence of tick-borne infectious diseases has increased worldwide in the recent decades, and ongoing climate change, human activity, mobility, bird migration and expanding tick habitats, will further increase risk areas [2]. The life span of the *Ixodes ricinus* ticks can last between two and six years, much longer than other vectors. Ticks can also survive harsh conditions through the ability to diapause. To pass each of three developmental stages ticks require a blood meal on a different host, thereby facilitating both the acquisition and transmission of infectious agents. In addition, *I*. *ricinus* has a very broad host specificity and can exchange pathogens with other tick species by co-feeding on a common host. In Switzerland, tick-borne encephalitis virus (TBEV) is the most important tick-borne virus; it can cause tick-borne encephalitis (TBE), a severe neurological disease [3, 4]. TBEV is a member of the genus flavivirus within the *Flaviviridae* family, and together with the Powassan virus (POWV), Louping ill virus (LIV), Langat virus (LGTV), Omsk hemorrhagic fever virus (OHFV), Kyasanur Forest disease virus (KFDV), and Alkhurma virus (ALKV) belongs to the mammalian group of tick-borne flaviviruses [3]. Based on genomic data, TBEV can be divided into three subtypes which differ in pathogenicity, clinical manifestation, and primary tick vector. These subtypes include the Western European TBEV (TBEV-EU), which is transmitted mainly by *I*. *ricinus* ticks and can cause death in 1–2% of the cases, the Siberian TBEV (TBEV-SIB) and the Far Eastern TBEV (TBEV-FE), which are transmitted mainly by *I*. *persulcatus* and can cause death in 6–8% and 30% of the cases, respectively [4–6]. Two other subtypes have recently been identified in the Himalayan and the Baikal regions [7–9]. The number of identified members of mammalian tick-borne flaviviruses has increased in recent years [10]. Furthermore, a new group of flavivirus-related viruses, the Jingmenvirus group (JMVG), was identified and classified in China in 2014 [11, 12]. Like other flaviviruses, members of the JMVG are enveloped particles with single-stranded (ss), positive-sense RNA genomes. However, while the genomes of the Jingmenviruses consist of four or five segments, the genomes of all other flaviviruses are non-segmented. The similarity between the JMVG and the other flaviviruses is limited also on the nucleotide sequence level, as only the non-structural protein genes NS5 and NS2B/NS3 are conserved [11, 13]. JMVG members have been detected in various arthropods and vertebrates worldwide [11, 14–16]. For example, Alongshan virus (ALSV) was first isolated in 2017 in China from blood samples of patients with febrile illness and a history of tick bites [13, 17]. While these patients showed clinical sings of TBE, neither TBEV RNA nor antibodies were detected. ALSV has also been detected in *I*. *persulcatus* ticks and mosquitoes from the same region [17] and subsequently in *I*. *ricinus* ticks from Finland and France, in *I*. *persulcatus* from Russia and in *Ixodes sp*. from Germany [12, 13, 18, 19].

The ALSV genome consists of four segments encoding two non-structural proteins NSP1 (flavivirus NS5-like) and NSP2 (flavivirus NS2B/NS3-like), the structural glycoproteins VP1a and VP1b, the nucleoprotein VP2, the membrane protein VP3, and an additional open reading frame designated nuORF or VP4 that overlaps with the VP1 coding sequence [11, 19, 20]. The widespread distribution and presumed pathogenicity of ALSV makes it a growing public health concern and highlights the importance of monitoring these pathogens to ensure that diagnostic, therapeutic, and preventive interventions are adapted.

In addition to viruses, bacteria and protozoa can be transmitted by *I*. *Ricinus* as well. For example, *Borrelia burgdorferi sensu lato* is a group of closely related gram-negative bacteria that cause Lyme borreliosis (LB), the most common tick-borne disease in the USA and Europe [21, 22]. This multisystemic infectious disease is transmitted to humans and animals by *Ixodes* ticks and often begins with a local erythema migrans at the bite site. If left untreated, the bacteria can spread and affect other organs i.e., the musculoskeletal system, the nervous system, the heart, and the eyes [23]. LB is not a notifiable disease in Switzerland, but it is recorded by the Federal Office of Public Health (FOPH) through a sentinel surveillance system. Since 2008, between 5300 and 16700 cases have been reported annually [24]. Previous studies have shown a prevalence between 19% and 26.54% in Swiss ticks [25–27].

The order *Rickettsiales* is another important group of bacteria with zoonotic potential that can be transmitted by ticks. Among them, *Rickettsia spp*. such as *R*. *helvetica*, *R*. *monacensis*, *R*. *conorii*, and *R*. *slovaca* are obligate intracellular, gram-negative bacteria which can be transmitted to humans by hard ticks and cause disease [28–31]. In Switzerland, *R*. *helvetica*, *R*. *monacensis* and *R*. *slovaca* are the most common species [32–34]. Clinical manifestations of rickettsiosis include non-specific symptoms such as fever and malaise, as well as exanthema, inoculation scab, lymphadenopathy, and endocarditis [35, 36]. *Anaplasma* and *Ehrlichia spp*. can cause an often self-limiting febrile illness or more severe human granulocytic anaplasmosis (HGA) or human granulocytic ehrlichiosis (HGE), respectively, which can be fatal in immunocompromised patients if untreated [36–39]. *Neoehrlichia mikurensis* (previously known as *‘Candidatus’ neoehrlichia mikurensis*) is another member of the *Rickettsiales* present in ticks and rodents in most European countries and in Asia [37–39]. Due to its tropism for endothelial cells, *Neoehrlichia mikurensis* has the potential to cause systematic inflammatory infections, particularly in immunocompromised individuals or those with comorbidities [40–42].

*Francisella tularensis* is a facultative intracellular bacterium which can cause tularemia in humans and animals, almost exclusively in the northern hemisphere, but several cases have been detected in Tasmania as well [43]. Small mammals such as rabbits (*Oryctolagus cuniculus*) and voles (*Myodes*) are particularly important as reservoir species. *F*. *tularensis* is divided into four subspecies i.e., *tularensis*, *holarctica*, *mediasiatica* and *novicida*, although there is some disagreement over the classification [44]. The subspecies differ in virulence and geographical distribution, with *holarctica* and *tularensis* having the greatest clinical relevance [45, 46]. Several major endemic areas exist in Europe, including Scandinavia, Western Russia, Czechia, and parts of Austria [47]. Transmission routes include ingestion of contaminated food or water, handling of infected animals, or bites by hematophagous arthropod vectors. In Switzerland, the disease has been notifiable since 2004, and according to the FOPH most of the cases have been transmitted by ticks. Between 2010 and 2016, the average number of reported cases was 0.43 per 100’000 inhabitants. In the last three years, the rate has more than quadrupled to 1.8 (range 1.4–2.5) per 100,000 [48]. Early signs of the disease include flu-like symptoms such as fever, fatigue, chills, and headache. Depending on the route of bacterial entry, several clinical forms of the disease can be differentiated [46].

*Babesia sp*. are intraerythrocytic protozoan parasites and the causative agents of babesiosis, an important emerging infectious disease worldwide [49]. It is a multisystemic disease with a spectrum of manifestations ranging from subclinical disease to severe illness and death [50]. In particular, splenectomised individuals are at high risk of fatal outcome [36, 51]. Tick-borne transmission is the primary route of *Babesia sp*. transmission, although blood transfusion, organ donation and vertical transmission in humans and animals have been reported as well [49, 52]. Seroprevalence studies identified *B*. *microti* and *B*. *venatorum* as the most common babesia species in Europe [53–55].

## Material and methods

### Sample collection

**Fig 1:**
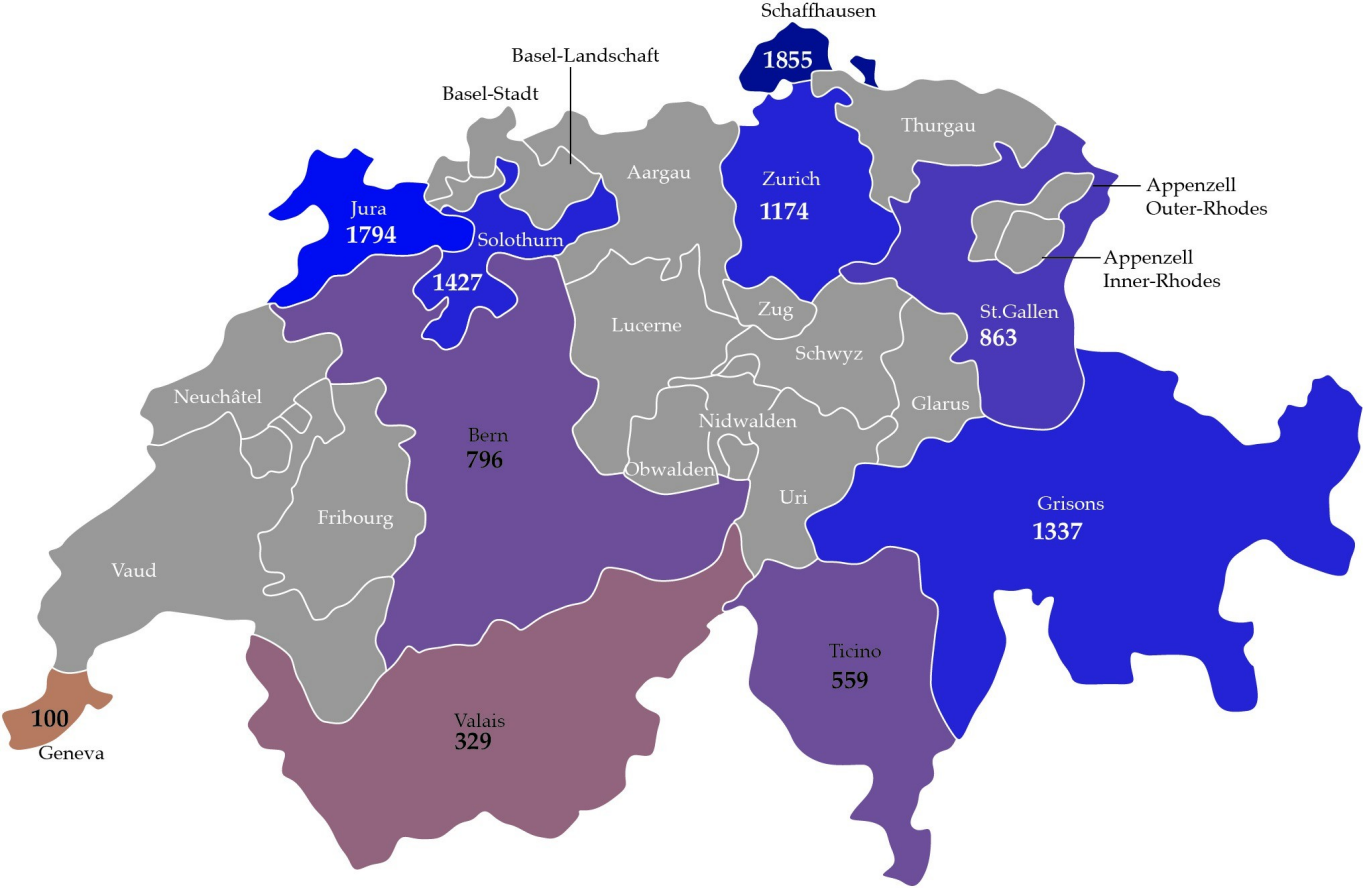
Map of Switzerland. Cantons where ticks were collected are shown in colors, and the total number of ticks is indicated. The continuous color scale represents numbers of ticks collected from low (light color) to high (dark color). Cantons where no tick collection took place are shown in grey.

Questing ticks were collected in urban/suburban and rural areas in ten Swiss cantons i.e., Solothurn (SO), Bern (BE), Geneva (GE), Valais (VS), Ticino (TI), Grisons (GR), Jura (JU), St. Gallen (SG), Schaffhausen (SH), and Zurich (ZH) (Fig 1). Specific places of collection were selected based on information from the App “Tick prevention” available for smartphones. This app was developed for Switzerland and Lichtenstein and allows users to report bites and sightings. Urban/suburban spots were defined as forests and parks with high human activity, within or close to main cities, while rural spots were defined as forest trails with rare human activity, outside of densely populated areas (S1 Table). The collection took place in May and September in 2021 and 2022, by flagging a 1m^2^ fabric over the ground on both sides of a footpath over a distance of 150 meters, resulting in 300 m^2^ of sampling area at each collection site. Ticks were stored in collection tubes and kept on ice until the end of the day, then killed by freezing, and stored at -20°C until further processing. In addition to the questing ticks, we examined 52 engorged ticks collected from roebucks and sika deer by hunters from canton SH. All ticks from an individual animal were pooled, resulting in 9 pools with 2 to 13 ticks each.

### Sample processing

Ticks were identified to the species level according to the identification guide by Estrada-Peña [56], washed twice with 70% ethanol, dried on a paper towel, and pooled in 2 ml Eppendorf tubes according to collection site, developmental stage, species, and gender (a maximum of 15 adult females, 15 adult males, or 50 nymphs per pool). To each pool, 650μl of Dulbecco’s phosphate buffered saline (DPBS, St. Louis, Missouri, USA) and a stainless-steel bead (5mm, QIAGEN, Hilden, Germany) were added, and the samples were mechanically homogenized in a TissueLyser II (Qiagen, Hilden, DE) at 30 Hz for 5 min. After centrifugation for 6 min at 16’060 x g (Biofuge Fresco, Heraeus, Hanau, DE), the supernatants were divided into aliquots of 200μl, 150μl and 230μl for the detection of non-viral pathogens by PCR, the detection of TBEV and ALSV by RT-PCR, and the assessment of the complete virome by next generation sequencing (NGS), respectively.

### DNA extraction and detection of non-viral pathogens by PCR

DNA extraction for detection of non-viral pathogens by PCR was performed using the ThermoScientific™ GeneJet Genomic DNA Purification Kit (Fisher Scientific, Massachusetts, USA) according to the protocol for DNA purification from cultured mammalian cells provided by the supplier, with the following modifications: Step 1 was skipped, and the protocol started with the addition of 200μl lysis solution and 20μl proteinase K solution to 200μl of the supernatant from the tick homogenates (see above). This was followed by the steps described for reduced volumes of elution buffer (100μl) in the supplier’s manual. As a control for DNA extraction, the tick mitochondrial 16S rRNA gene was amplified by qPCR using tick-specific primers as previously described [57]. *Babesia spp*., *Ehrlichia spp*., *B*. *burgdorferi sensu lato*, and *Rickettsia spp*. DNA was detected using specific alphaCube qPCR kits (Mikrogen GmbH, Neuried, Germany) with settings according to instructions provided by the supplier. To detect *F*. *tularensis* and *N*. *mikurensis* DNA, we used primer sequences and PCR conditions described in previous studies [27, 58]. The Institute of Medical Microbiology (University of Zurich, Switzerland) kindly provided positive control DNA for *Francisella* and *Neoehrlichia*. For qPCR preparation the QuantiNova Pathogen + IC Kit (QIAGEN GmbH, Hilden, Germany) was used according to the manual, except that half the amount of each reagent and no ROX was used. All qPCRs were run on the QuantStudio™ 3 Real-Time PCR System (Fisher Scientific, Massachusetts, USA) except for alphaCube Borrelia/Rickettsia, which was run on QuantStudio™ 7.

### RNA extraction and detection of TBEV and ALSV by RT-qPCR

RNA was extracted from supernatants of tick homogenates using the QIAamp Viral RNA mini kit (QIAGEN GmbH, Hilden, Germany) according to the supplier’s manual, except that carrier RNA was omitted. TBEV RNA was identified by using the alphaCube TBE kit (Mikrogen GmbH, Neuried, Germany) according to the provided protocol and performed on a QuantStudio™ 3 Real-Time PCR System (Fisher Scientific, Massachusetts, USA) with the following settings: 20 min at 45°C for the reverse transcription, 5 min at 95°C, and 45 cycles of 10 s at 95°C, 20 s at 60°C, and 10 s at 72°C. ALSV RNA was identified using the QuantiNova Pathogen + IC kit (QIAGEN GmbH, Hilden, Germany) according to the provided protocol, with primers and probe published by Wang et al. [17].

### Sample preparation for NGS

Supernatants from tick homogenates were first enriched for virus particles as described previously [59]. Then, total RNA and DNA were extracted using the QIAmp Viral RNA mini kit (Qiagen GmbH, Hilden, Germany) according to the instructions, except that no carrier RNA was used and 1% β-mercaptoethanol (Bio-rad, Hercules, California, USA) was added to the AVL lysis buffer to inactivate the nucleases used for the virus enrichment step. To reduce the total number of samples to be sequenced, pools were re-pooled as follows: the extracted nucleic acid from up to 6 pools of ticks that matched concerning collection site, developmental stage, sex, and species was mixed in equal parts to a total volume of 30μl. This resulted in NGS pools containing nucleic acid from a maximum of 90 females, 90 males or 250 nymphs. If less ticks of the same developmental stage were available, adults and nymphs of the same collection site were mixed. Reverse transcription, second strand synthesis, and random amplification was performed as previously described using the SISPA (sequence-independent single-primer-amplification) method [59]. DNA was sheared by sonication (E220 Ultrasonicator, Covaris, USA) at the Functional Genomics Center Zurich (FGCZ, Zurich, Switzerland), targeting fragments of 500bp length. Sequencing libraries were made using the NEBNext Ultra II DNA Library Prep Kit for Illumina (New England Biolabs, Ipswich, MA, USA), cleaned up with AMPure XP beads (Beckman Coulter, Brea, CA, USA), and barcoded using NEBNext Multiplex Oligos (96 Unique Dual Index Primer Pairs; New England Biolabs, Ipswich, Massachusetts, USA). To measure the molarity of the libraries and the fragment size distribution, the Agilent 2200 TapeStation was used with D1000 HS- and D1000-ScreenTapes (Agilent Technologies, California, USA). Finally, libraries were sequenced in a paired-end NGS run with 2 x 100 or 2 x 150 nucleotides read length, on an Illumina NovaSeq 6000 Benchtop sequencer (Illumina, San Diego, CA, USA) at the FGCZ. PhiX Control v3 Library (Illumina, California, USA) was used as sequencing control.

### Data analysis

The generated sequences were analyzed by *de novo* assembly and reference-based assembly pipelines as described previously [59–61]. Using Trimmomatic (version 0.36) and cutadapt (version 3.2), SISPA primers (“-b GTTGGAGCTCTGCAGTCATC -B GTTGGAGCTCTGCAGTCATC”), low-quality sequencing ends, and the sequencing adapters were removed. Then, quality-checked reads were assembled using metaspades (v3.12.0) and generated *de novo* contigs were compared to the NCBI non-redundant database (nt) using blastn (v2.10.1+) [62]. Additionally, quality-controlled reads were aligned using a metagenomic pipeline of the SeqMan NGen v.17 software (DNAStar, Lasergene, Madison, WI, USA) to an in-house database containing over 60’000 full-length virus genomes and genomes reported from “tick” as a host downloaded from the NCBI database. The viral contigs and selected coding regions of TBEV and ALSV were further investigated and aligned using MUSCLE in MEGA 11, and phylogenetic trees were constructed in Mega X using the Maximum Likelihood algorithm with 1000 bootstrap values and a cut-off of 70% [63]. Similarities between contigs were calculated by percent identity matrix in Clustal2.1 (https://www.ebi.ac.uk/Tools/msa/clustalo/).

### Statistical analysis

All analyses were undertaken in R [64]. An estimate of the viral and bacterial burden was made by taking the inverse of the CT (cycle threshold). Negative CT values were assigned as 0. A tweedie generalised linear model was used to analyse the data with the viral load a continuous dependent variable with an excess of 0s [65]. This was achieved by using the family tweedie provided by the statmod library [66]. The viral and bacterial burden in the tick pools was analyzed using a backward stepwise regression analysis according to the explanatory variables of canton of origin, number of ticks in the tick pool, season the ticks were collected, and whether the ticks originated from an urban or rural area. A p value of <0.05 was considered significant.

### Accession numbers

The generated contigs of TBEV and ALSV from this study have been deposited in the NCBI database under GenBank accession numbers OQ555296 to OQ555317. The raw reads have been deposited under BioProject accession number PRJNA906035 and BioSample accession numbers: SAMN31890811 and SAMN36735771- SAMN36736013.

## Results

### Tick collection

During the four time points over two consecutive years a total of 10’286 ticks of the species *I*. *ricinus* were collected, of which 10’234 (1’631 females, 1’763 males and 6’840 nymphs) were collected by flagging method and 52 (34 females, 5 males and 13 nymphs) were collected from hunted wild ruminants. Considerable heterogeneity in the numbers of ticks collected at the different sites was observed between the different cantons, the different collection time points (May versus September), and the different sites (urban/suburban versus rural).

Fig 1 and Table 1 show an overview of the total numbers of ticks collected in the different cantons (Fig 1 excludes ticks from hunting). The highest numbers were from SH and JU, with 1’855 and 1’794 ticks respectively, while the lowest numbers were from GE and VS, with 100 and 329 ticks, respectively. However, as shown in S1 Table, in cantons SH, SG and ZH, ticks were collected at three sites, resulting in 900m^2^ of sampling area each, while in all other cantons only two sites were sampled per time point (600m^2^). Although S1 Table indicates three collection sites also for SO, only two sites were sampled per time point, but due to deforestation a new rural site, located several hundred meters away from the original site, had to be chosen the second year. In both years, the number of ticks collected in September was lower (429 and 756 ticks) than in May (4’259 and 4’790 ticks). Overall, the 10’286 ticks were divided into 448 pools for PCR analysis and 242 pools for NGS.

**Table 1:** Overview of the numbers of collected ticks and numbers of PCR- and NGS pools.

Collection timepoint	Total number of ticks	Min./max. number of ticks per sites	Number of PCR pools	Number of NGS pools
May 2021	4’259	13/1’000	163	70
September 2021	429	0/155	35	35
May 2022	4’790	5/1’058	186	73
September 2022	756	0/125	55	55
Hunt 2021	52	2/14	9	9
**Total**	**10’286**	**0/1’058**	**448**	**242**

### Selected non-viral pathogens

Tick pools were screened by PCR for selected non-viral pathogens as described in Materials and Methods. Overall, from 439 tick pools, 339 (77.2%) were positive for at least one of the selected non-viral pathogens. The highest pool positivity for all non-viral pathogens investigated was in May 2021 with 84%, followed by 79% in May 2022, 67.3% in September 2022 and 51.4% in September 2021. Of all pools, 28.9% were positive for at least two different pathogens, with the most common combination being *Ehrlichia spp*. and *Rickettsia spp*. Across all four collection time points, 83.9% of urban pools were positive for at least one non-viral pathogen, compared to 70.6% of rural pools. Surprisingly, despite the very few ticks collected, GE had the highest diversity of pathogen occurrence, with rural and urban pools from May 2021 simultaneously positive for four of the pathogens, i.e., *Borrelia*, *Rickettsia*, *Neoehrlichia* and *Ehrlichia*. The results are shown in detail in Table 2 and S2 and S3 Tables and can be summarized as follows (data from engorged ticks are excluded from statistical analysis, due to the small sample number):

**Table 2:** Pool positivity (%) and range of CT values for selected non-viral pathogens.

	*Rickettsia sp*.		*Ehrlichia sp*.		*Borrelia sp*.		*Neoehrlichia mikurensis*		*Babesia sp*.		*Francisella tularensis*	
**Collection timepoint**	%	CT	%	CT	%	CT	%	CT	%	CT	%	CT
May 2021	79.8	21–35	39.9	24–40	5.5	28–39	2.5	31–39	0.6	38	0.0	-
September 2021	40.0	22–30	40.0	26–39	0.0	-	5.7	33–36	0.0	-	0.0	-
May 2022	73.7	20–34	35.5	31–40	2.2	32–38	0.0	-	0.0	-	0.0	-
September 2022	56.4	21–36	25.5	28–40	10.9	31–38	0.0	-	0.0	-	1.8	32
Hunt 2021	55.6	25–32	55.6	28–39	0.0	-	0.0	-	0.0	-	0.0	-
**Total**	**70.8**	**20–36**	**36.6**	**24–40**	**4.2**	**28–39**	**1.3**	**31–39**	**0.2**	**38**	**0.2**	**32**

*Rickettsia spp*. showed the highest pool prevalence (40–80%) and the lowest CT values (20–36). All cantons had repeated positive pools, especially SO and ZH, where *Rickettsia* was detected at every collection timepoint; GE showed the least pool positivity. Five out of 9 pools of engorged ticks were also *Rickettsia spp*. positive. Tick pools from urban sites were more positive compared to rural areas (p = 0.02, S4 Table), and the number of pathogens correlated positively with the number of females (p = 0.003, S4 Table) and nymphs (p<0.0001, S4 Table).

*Ehrlichia spp*. had the second highest pool prevalence (25–40%) and was detected at least once in all cantons, but most frequently in rural and urban areas of the cantons SO, GR and ZH. Interestingly, we observed a higher load of *Ehrlichia* in ticks from 2021 than in ticks from 2022 (p<0.001, S4 Table) and a positive correlation between number of nymphs and bacterial load (p<0.001, S4 Table).

*B*. *burgdorferi sensu lato* was detected in all cantons except TI. ZH pools were most frequently positive for *Borrelia*, i.e., at 3 out of 4 sampling times. The only statistically significant difference was observed between rural and urban ticks. Thus, the probability that a tick is carrying *Borrelia* bacteria seems to be higher in urban areas (pool positivity 8.2%) than in rural areas (pool positivity 1.9%; p = 0.016, S4 Table).

*N*. *mikurensis*, *F*. *tularensis* and *Babesia sp*. were detected far less frequently than any of the other selected pathogens. Specifically, six pools were positive for *Neoehrlichia*, all of which originated from 2021. These included four pools collected in May 2021 (three pools of nymphs and one pool of male ticks) in the cantons of BE, GE, and VS, and two nymph pools collected in September in the cantons of ZH and JU. Out of 439 pools, only a single pool of nymphs collected in an urban area of canton Bern was positive for *Francisella*, and only a single pool of adult ticks collected in an urban area of canton GE was positive for *Babesia*.

Of the nine pools of ticks collected from game in canton SH, five pools were positive for *Ehrlichia* and *Rickettsia* (55.6%). However, due to the low numbers of ticks and pools, no statistical analysis was performed.

### The virome of Swiss ticks

**Fig 2:**
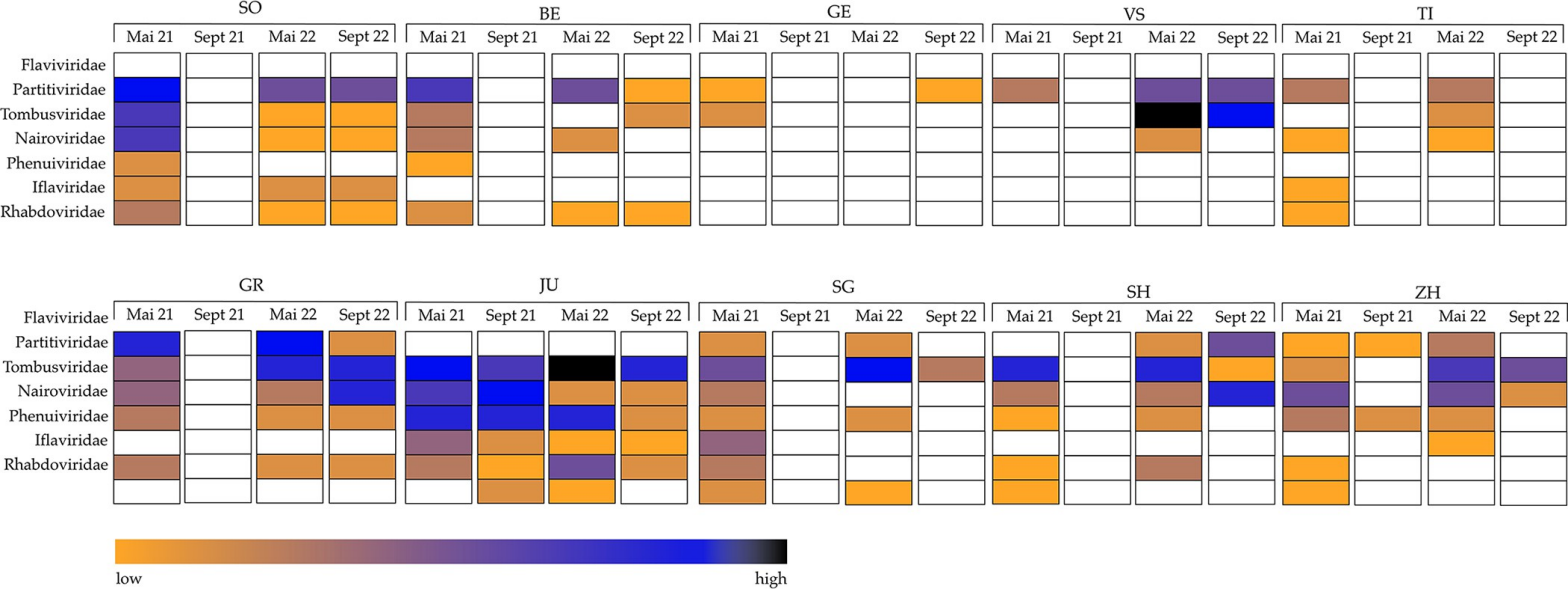
Overview of virus families detected at the four collection timepoints in the ten cantons. The heatmap represents the reads per million from low to high. White = not detected.

To assess the virome of the ticks a total of 9.17 × 10^9^ sequencing reads were generated, with an average of 3.94 × 10^6^ reads per pool sample (range from 3 × 10^5^ to 2 × 10^7^). The analysis revealed genome sequences of viruses from seven different families, i.e., *Flaviviridae*, *Partitiviridae*, *Tombusviridae*, *Nairoviridae*, *Phenuiviridae*, *Iflaviridae and Rhabdoviridae* (Fig 2). Most frequently and in all cantons, sequences from *Partiti*- and *Tombusviridae* were identified. However, in cantons SG, TI, and GE, *Tombusviridae* were detected only at a single timepoint. Sequence contigs showed up to 100% identity to Norway partiti-like virus 1 and Norway luteo-like virus 1 and 3, which were previously detected in *I*. *ricinus* ticks from Norway [67]. *Nairoviridae* sequences were abundant, especially in the May collections, and were detected in all cantons except GE. The highest read numbers were from the canton JU for all collection time points, followed by ZH, GR and SO, where *Nairoviridae* sequences were identified at three of the four timepoints. Samples from SG, BE, TI, and SH tested positive for *Nairoviridae* exclusively in the May collections. In canton VS, nairovirus sequences were only detected at one time point, specifically in May 2022. Generated contigs showed approximately 98% identity to unclassified Norway nairovirus 1 found in *I*. *ricinus* in Norway and 98% identity to Grotenhout virus detected in *I*. *ricinus* in Belgium [67, 68]. *Rhabdoviridae* sequences were detected at three timepoints in BE and SO, at two timepoints in cantons SG and JU, and once in the cantons of ZH, TI and SH. The samples from all other cantons tested negative. Contigs of rhabdovirus sequences showed approximately 92% identity to Norway mononegavirus, an unclassified rhabdovirus first described in *I*.*s ricinus* from Norway [67]. *Phenuiviridae* sequences were detected least frequently, except for canton JU, where samples from all timepoints tested positive. In ZH, SO, SG, and Bern *Phenuiviridae* sequences were identified at one timepoint, in GR, VS, TI, SH, and GE at no time point. Sequences showed up to 99.9% identity to Norway phlebovirus 1 which was first described in *I*. *ricinus* in Norway [67]. *Iflaviridae* sequences were found in seven of the ten cantons selected for the study, most frequently in JU, with samples from all four collection time points testing positive, followed by GR and SO, where only in September 2021 no *Iflaviridae* sequences were found. In SH samples from two timepoints, and in cantons ZH, SG and TI samples from a single timepoint were positive. BE, VS and GE remained negative for *Iflaviridae* sequences over the entire study period. The detected sequences showed approximately 99% identity to Iflavirus IricIV-2 which was already described in a *I*. *ricinus* lab strain from Neuchâtel, Switzerland [69]. *Flaviviridae* sequences were found in tick samples from six of the ten cantons, first and foremost in GR at three of the four collection time points, followed by ZH, SG and SH. Specifically, samples from both May collections in ZH and SG, and from both 2022 collection timepoints in SH tested positive. Most importantly, in addition to TBEV, we identified ALSV sequences for the first time in Switzerland, both in questing and engorged ticks [70]. Since TBEV and ALSV are of particular interest due to their zoonotic potential, we carried out phylogenetic analyses.

**Fig 3:**
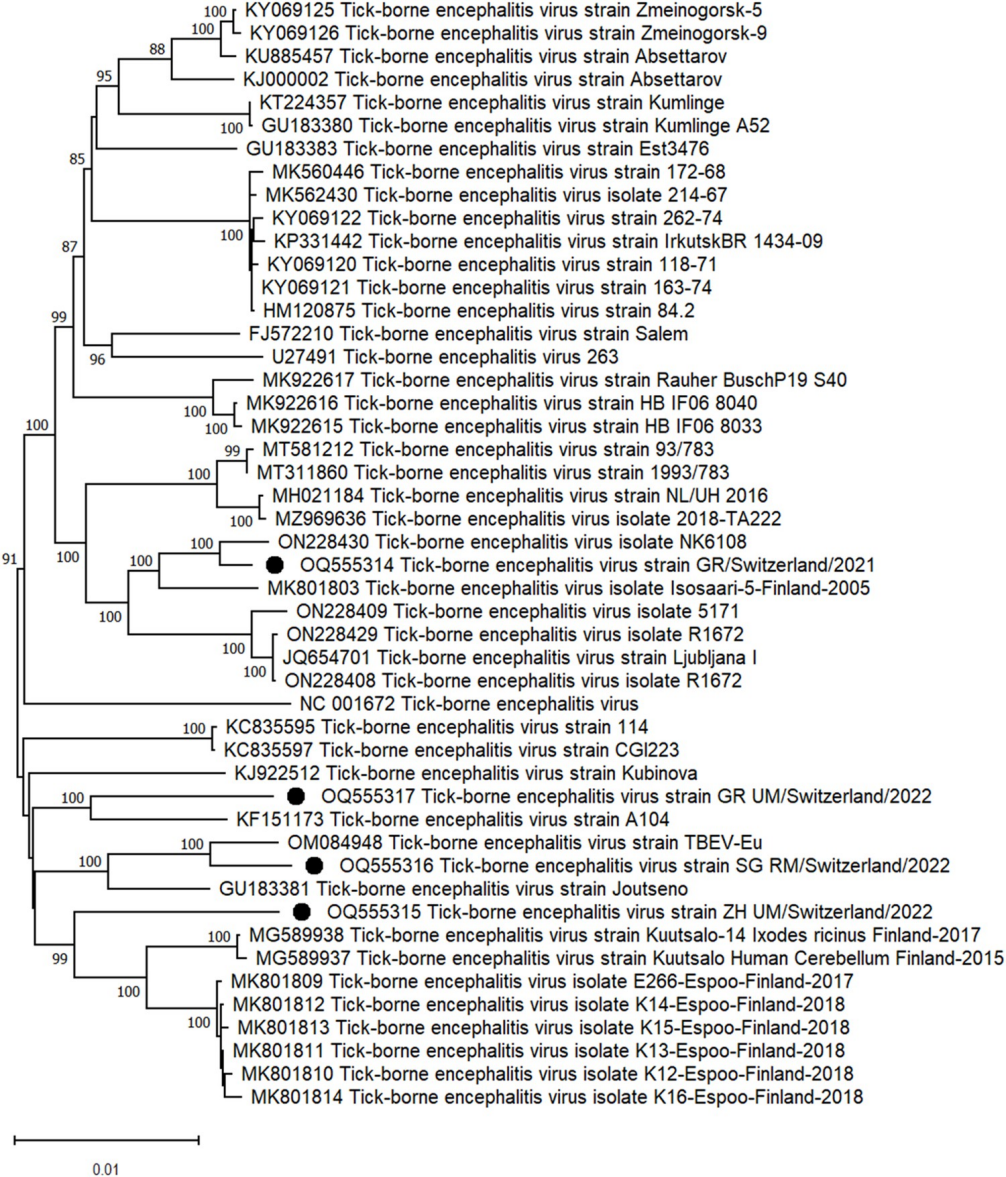
Phylogenetic tree based on amino acid sequence identity of the polyprotein of selected TBEV genomes. Dots: strains detected in this study. Phylogenetic analysis was performed using the maximum likelihood algorithm based on the Tamura–Nei model with the 1000 replication bootstrap method using MEGA 11 software. Only values ≥ 70% are displayed.

Four complete or nearly complete TBEV protein-coding sequences were assembled all of which belong to the EU subtype. Strain GR/Switzerland/2021 (OQ555314), sequenced from adult female ticks collected in a rural area of the canton GR, showed 99.5% nt identity to strain NK6108 from Slovenia (ON228430). The second TBEV protein-coding sequence, GR_UM/Switzerland/2022 (OQ555317), which originated from urban male ticks, showed 97% nt identity to the GR strain from the previous year (OQ555314) and 98.3% nt identity to the A104 strain from Austria (KF151173). TBEV sequences from urban male ticks in canton ZH, ZH_UM/Switzerland/2022 (OQ555315), showed 98.2% nt identity to strain E266-Espoo-Finland (MK801809). Finally, the strain SG_RM/Switzerland/2022 (OQ555316), sequenced from male ticks collected in a rural area of canton SG, showed 99% nt identity to strain TBEV-Eu from Italy (OM084948). Although all 4 generated TBEV genomes were sequenced from ticks collected from neighbor cantons differences were found and all clustered separately (Fig 3).

**Fig 4:**
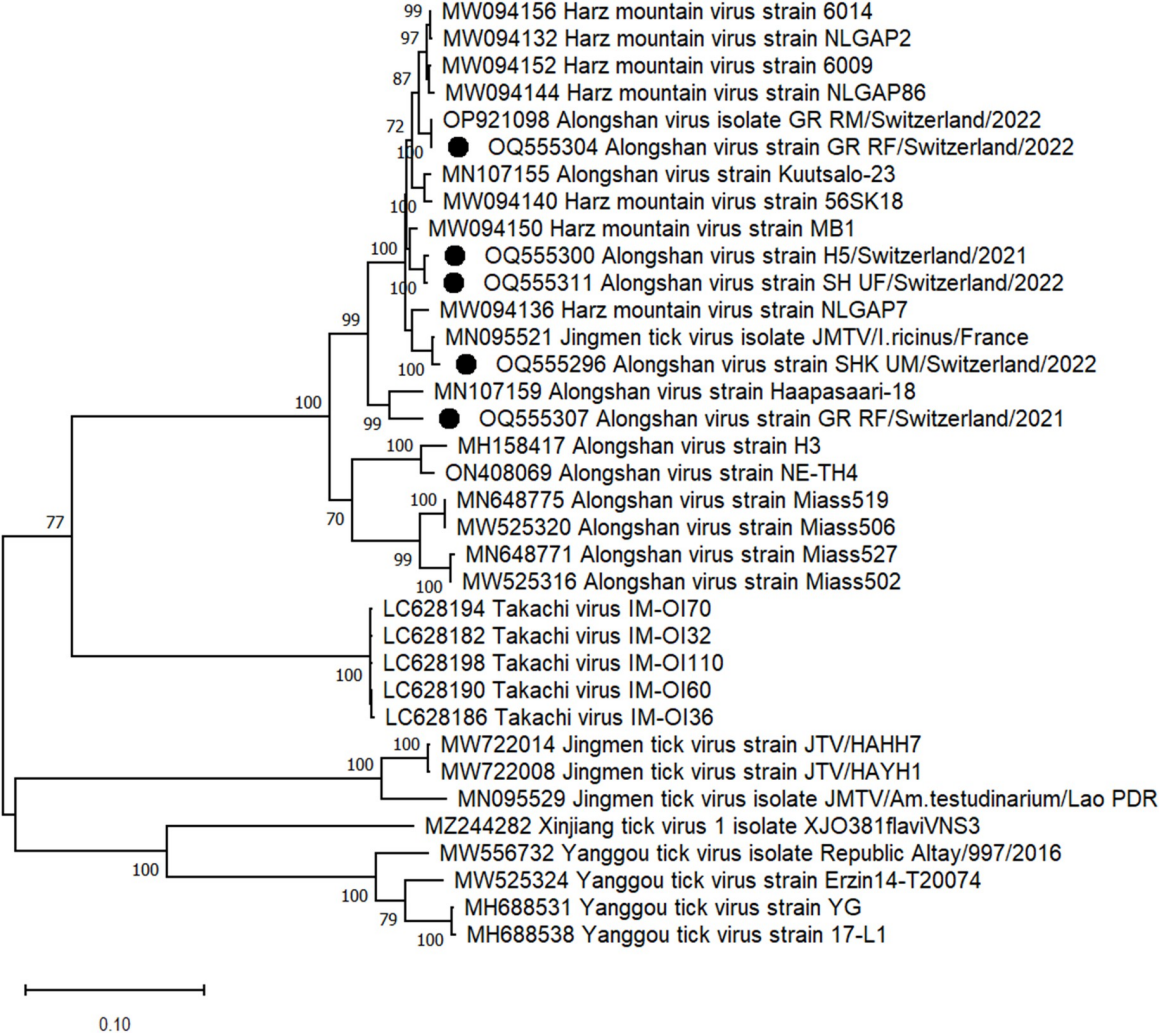
Phylogenetic tree based on amino acid sequence identity of the NS3 protein of selected flavi-like viruses. Black dots: ALSV strains detected in this study. Phylogenetic analysis was performed using the maximum likelihood algorithm based on the Tamura–Nei model with the 1000 replication bootstrap method using MEGA 11 software. Only values ≥ 70% are displayed.

**Fig 5:**
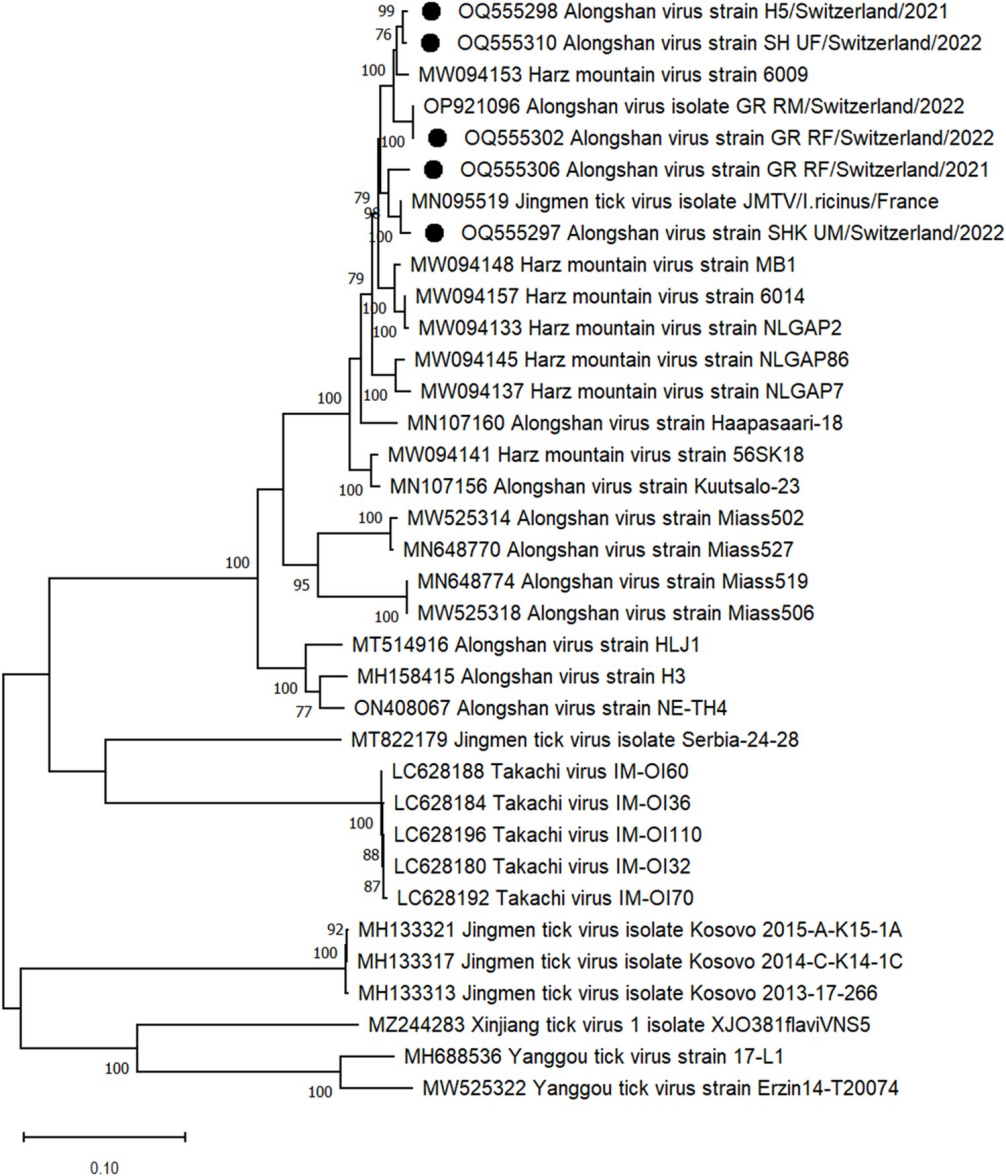
Phylogenetic tree based on amino acid sequence identity of the NS5 protein of selected flavi-like viruses. Black dot: ALSV strains detected in this study. Phylogenetic analysis was performed using the maximum likelihood algorithm based on the Tamura–Nei model with the 1000 replication bootstrap method using MEGA 11 software. Only values ≥ 70% are displayed.

We previously reported the protein coding sequence of an ALSV strain from the May 2022 collection in canton GR (OP921098) [70]. Here, we assembled the protein coding sequences of 5 additional ALSV genomes identified in ticks from 2 different cantons, i.e., GR and SH. As opposed to the TBEV sequences, the ALSV sequences clustered together with all other ALSV sequences previously found in ticks in Europe (Figs 4 and 5). Specifically, the generated ALSV contigs and the previously reported sequences showed 95.6–97.6% nt identity in segment 1, 91–97.9% in segment 2, 89–99.5% in segment 3, and 96.4–99.4% in segment 4. Interestingly, the highest similarity was found between contigs from female ticks collected in 2022 at the urban site of canton SH (SH_UF/Switzerland/2022) and ticks collected in 2021 from sika deer (H5/Switzerland/2021) in the same canton. ALSV contigs assembled from sequences obtained from urban male ticks collected in 2022 in canton SH (SHK_UM/Switzerland/2022) showed 99.4%, 99.5%, 99.6%, and 99.5% nt identity to the respective 4 segments of a Jingmen tick virus isolated from *I*. *ricinus* in France (MN095519- MN095522). Contigs generated from ticks collected in 2021 from sika deer (*Cervus nippon*) in canton SH (H5/Switzerland/2021) showed 98.6%, 98.9%, 97.8% and 99% nt identity to the respective segments of the Harz Mountain virus from Germany (MW094152-MW094155). The third ALSVs contigs assembled from female adult ticks collected in 2022 in an urban area of canton SH (SH_UF/Switzerland/2022) showed 98.7%, 98.7%, 97.9% and 99% nt identity to the same Harz Mountain virus strain. Interestingly the 4 segments assembled from rural female ticks from canton GR collected in 2022 (GR_RF/Switzerland/2022) showed 100%,100%, 100% and 99.9% nt identity to the contigs previously generated from male ticks collected in the same year and area (OP921096-OP921099). However, the contigs generated from adult females collected in the same area in 2021 i.e., GR_RF/Switzerland/2021 showed only 96.6%, 97%, 94.5% and 97.5% nt identity to the respective segments of the genome sequenced one year later i.e., GR_RM/Switzerland/2022 and GR_RF/Switzerland/2022, resulting in 5, 7, 1, 2, 1 and 3 amino acid substitutions in NS5-like protein, glycoprotein VP1a, glycoprotein VP1b, NS3-like protein, capsid protein, and membrane protein, respectively.

Table 3 and S2 and S5 Tables show CT values and/or RT-qPCR pool positivity for TBEV and ALSV. With some heterogeneity across the cantons, TBEV was detected in 19 out of 448 pools (4.24% including the pools of engorged ticks), confirming the results from sequencing. These were exclusively pools from cantons GR, SG, and ZH, which were positive at all four collection time points, with CT values ranging from 22 to 39, and a pool from a single timepoint (September 2022) from SH with a CT value of 36. Overall, there was no significant difference between urban and rural sites or developmental stages of the ticks; however, TBEV was more frequently found in 2022 than in 2021 (p = 0.02, S4 Table) which corresponds to an increase of human cases from 199 in 2021 to 264 in 2022 [24]. In SG, only pools from rural areas were positive for TBEV, while in ZH and SH it was only the pools from urban regions. The only canton with TBEV-positive pools in both rural and urban areas was GR. Overall, 5.5% (9/164) of nymph pools were positive for TBEV, followed by 4.3% (5/117) of female pools and 4.0% (5/124) of male pools (S5 Table).

**Table 3:** Pool positivity (%) and range of CT values for TBEV and ALSV.

	*TBEV*		*ALSV*	
Collection timepoint	%	CT	%	CT
May 2021	1.84	22–39	3.06	27–35
September 2021	0.00	-	5.71	30, 34
May 2022	6.99	22–34	11.29	17–38
September 2022	5.45	26–36	7.27	21–31
Hunt 2021	0.00	-	0.22	22, 26
**Total**	**4.24**	**22–39**	**7.59**	**17–38**

For ALSV, the pool-positivity was 7.59% (34 out of 448, including the pools of engorged ticks) with some variation between different cantons. The overall virus load was higher in 2022 than in 2021 (p = 0.006; Table 3 and S4 Table) and significantly higher in nymphs than in the adult pools (p<0.001; S4 Table). Specifically, the ALSV pool-positivity was 15.9% (26/164) for nymph pools, 2.4% (3/124) for male pools and 2.6% (3/117) for female pools. Cantons where ALSV was detected repeatedly include SG, SH, GR and ZH (S2 Table). Statistically the highest chance to find ticks with TBEV is in SG and ZH and ticks with ALSV in GR and SH (p<0.01 in all cases).

## Discussion

To analyze the risk potential of Swiss ticks, we carried out an extensive surveillance over a two-year period in multiple regions of the country. Yields of ticks varied greatly between the two different sampling timepoints May and September (9049 versus 1185 ticks), which is consistent with the registered human tick-borne diseases published by the FOPH [24]. There was also considerable variation between the different collection sites, which ranged from 0 to 1058 ticks. However, only a small difference was observed when comparing yields from rural and urban sites. Extrapolated to all collection sites, the frequency was 0.44 and 0.31 ticks per m^2^ in rural and urban areas, respectively, but these figures are subject to bias because we explicitly selected areas with a high risk for tick bites, according to the “Tick Prevention” app developed at the Zurich University of Applied Sciences ZHAW.

In addition to an unbiased sequencing approach to determine the entire virome of Swiss ticks, which was the main focus of our study, we also screened for specific non-viral pathogens. It must be emphasized, however, that comparisons between our results and the results from other studies, e.g., concerning the prevalence of specific pathogens, are difficult, because we analyzed pools rather than individual ticks.

The high pool positivity for *Rickettsia sp*. was rather unexpected. To date the highest rate previously reported was from ticks collected from a vegetation-rich dune area in the Netherlands (66%) [71]. The method we used can detect a wide range of *Rickettsia sp*., all of which are potentially pathogenic, but not all have been shown to be transmitted by ticks. In addition, *Dermacentor* and *Rhipicephalus* tick species have been reported as the main vectors for many species and *I*. *ricinus* ticks only rarely [72]. However, based on the high pool positivity for *Rickettsia*, *I*. *ricinus* may play a greater role as a vector than thought. A study from 2003 suggested that *Rickettsia* infections might be endemic in Switzerland, based on serological data from patients with febrile illness following a recent tick bite [73].

For *Anaplasmataceae*, a family within the order of *Rickettsiales* which includes *Anaplasma* and *Ehrlichia spp*., various European studies have reported prevalence rates in *I*. *ricinus* ranging from 0.4 to 66.7% [27, 74]. *Anaplasma phagocytophilum* is of particular importance, as anaplasmosis is one of the most common tick-borne diseases in animals in Europe [75, 76]. In Switzerland, 0.02% - 1% of the serum samples from dogs and foxes have tested positive for *A*. *phagocytophilum*, but neither *E*. *canis* nor *A*. *platys* were detected [77, 78]. In Italy, a serum prevalence of up to 17% for *A*. *phagocytophilum* has been reported in red foxes [79]. All these studies indicate the reservoir potential of red foxes, which are increasingly found in urban areas, but other species may play a role as well, as there are reports of *Ehrlichia sp*. in birds from South America and Hungary and in ruminants in China and Japan] [80–82]. Sequences obtained from Hungarian birds showed the highest identity to *E*. *chaffeensis*, the causative agent of human monocytic ehrlichiosis, which is emerging in the USA.

The high pool positivity observed in our study suggests that *Anaplasmataceae* members should not be ignored as tick-borne pathogens. In addition, pools with high numbers of nymphs, which are more difficult to be detected while feeding on humans, were found to have higher *Ehrlichia* loads. Compared to previous studies from Switzerland, which reported prevalences between 7.3 and 13.5% for *Rickettsia* and between zero and 1.4% for *A*. *phagocytophilum*, our pool positivity appears very high. However, this may be explained by the fact that we have detected more different *Rickettsia* species, thus more species may be endemic than previously thought [31, 83].

The presence of *B*. *burgdorferi sensu lato* was confirmed both in rural and urban ticks, as previously reported in other European countries [84, 85]. The white-footed mouse (*Peromyscus leucopus*) serves as a reservoir species for *B*. *burgdorferi sensu lato* and is concentrated in urban and suburban areas causing higher infection in ticks, which may explain the statistically higher prevalence in urban ticks in our study [86–88]. While nymphs appear to play a major role for the transmission of *B*. *burgdorferi sensu lato* [84], we did not observe a significant difference in the pool positivity between adults and nymphs.

Our study revealed a pool positivity for *Babesia sp*. of 0.2%. This appears low compared to previous studies that confirmed the presence of *B*. *microti*, *B*. *venatorum* and *B*. *divergens* in Swiss ticks at a prevalence of 0.7 to 1.7% [89]. However, *Babesia sp*. are known for large regional differences in prevalence in ticks, e.g., in Austria prevalence ranges from 0 to 100% in different regions of the country and in Germany from 1 to 10.7% [53, 90].

The low pool positivity for *F*. *tularensis* is consistent with previous studies in Switzerland, which have shown a prevalence of 0.02–0.12% [27, 91]. Interestingly, in both previous studies, the Bern region was listed as an area with a negligible risk for *F*. *tularensis*, and cases seem to come mainly from northeastern areas of Switzerland [92]. However, the only positive pool detected in our study was collected in the canton of Berne.

Consistent with the virome analyses of ticks from other countries [67, 93–96], the genomes of RNA viruses were more frequently detected than DNA viruses also in Swiss ticks. RNA viruses pose a particular threat, as their high mutation rate supports rapid adaptability and the establishment of enhanced virulence and tenacity. We detected a large variety of different virus families at all collection timepoints except for September 2021, when yields from tick collections were sparse except for canton JU. There virus variety was high at all timepoints (Fig 2).

By far the most frequently detected viral reads belonged to both *Tombus*- and *Partitiviridae*, which are mostly known to be associated with plants and fungi, and in the case of *Partitiviridae* also with protozoa. The fact that other virome analyses show similar results for *Tombusviridae* and *Partitiviridae* makes it unlikely that these viruses originate from environmental contamination; their high frequency supports the assumption that these indeed are tick-associated viruses, as previously suggested by Pettersson [67, 97].

The *Bunyavirales*, which include the families *Nairo*- and *Phenuiviridae*, were the second most abundant, although the *Phenuiviridae* were significantly less abundant than the *Nairoviridae*. *Bunyavirales*, have a genome with three ssRNA segments (S, M, L). The S segment encodes for the nucleocapsid protein, the M segment encodes the glycoprotein, and the L segment encodes the RNA-dependent RNA polymerase. Crimean Congo haemorrhagic fever virus, which is widespread in Africa, Asia as well as Southern and Eastern Europe, and Nairobi sheep disease virus in Africa and India, both orthonairoviruses, are the best-known members of the *Nairoviridae* with implications for animal and human health [96, 98, 99]. *Phenuiviridae* consist of 20 different genera of which the phleboviruses are spread worldwide and mainly transmitted by arthropods, especially mosquitoes [65, 66]. Important examples include the Rift Valley Fever Virus and the Toscana virus, and the list of newly discovered members is constantly growing [100]. Virome analyses from Norway suggest that these common virus families may be tick-associated [67], as do our data with high detection frequencies in widely separated areas. Recent metavirome studies from China have shown segment or gene loss mainly in tick-specific clades in *Phenui*- and *Nairoviridae* [99]. Data from our study support this theory as we have readily detected sequences from the S and L segments but not the M segment.

The *Rhabdoviridae* is a diverse family of plant and animal viruses, which includes important pathogens such as rabies virus and vesicular stomatitis virus, members of the genera Lyssavirus and Vesiculovirus, respectively [100]. *Rhabdoviridae* have already been detected in several virome studies of ticks not only in Europe but also in Asia [67, 97, 101, 102] and even with higher frequencies than in our study.

The *Iflaviridae* is a family of viruses that infect arthropods and do not pose a threat to humans or animals but may offer an interesting approach to arthropod control. It has been shown that some *Iflaviridae* can negatively affect the fitness of their vector hosts, which offers a potential strategy for vector control [103–105]. In general, a very similar pattern of *Iflaviridae* composition seems to emerge for *Ixodes sp*. with some variations between different countries, which is another possible indication of tick association [67, 93, 97, 106].

In both years, we detected TBEV sequences in ticks collected from several sites, including places where TBE cases have already been reported [107]. According to the FOPH, between 200 and 450 cases of TBE occur annually, and the number of cases has been increasing for the past five years. According to the annual TBEV report of the European Centre for Disease Prevention and Control, 3,734 confirmed TBE cases occurred in EU/EEA countries in 2020 (0.9/100,000 inhabitants), more than in 2019 (0.7/100’000 inhabitants) and more than 2016–2018 (0.6/100’000 inhabitants) [24, 108]. Previous studies reported a TBEV prevalence in *I*. *ricinus* ticks of 0.1–5% in Germany, 0–9% in Norway (with higher rates in adult ticks), 1.6% in Eastern Poland (10.8% in *Dermacentor reticulatus*), 0.11% in France, 0% to 11.11% in Switzerland, and 1.2% in Northern Italy [7, 31, 109–115]. While not directly comparable as we are reporting pool positivity, our numbers are in the same range. Rieille et al. [114] detected TBEV-positive ticks along the Rhône River in the canton VS. Our collection sites in VS were further west, and we did not detect any shift or expansion of these confirmed TBEV foci. Although adult ticks ingest more blood meals which would suggest a higher prevalence, as they are capable of effective transstadial transmission [116], TBEV was not more prevalent in adult ticks than in nymphs.

The TBEV protein-coding sequences reported here belong to the TBEV-EU subtype and are consistent with the TBEV-subtyping performed in previous Swiss studies [114]. Although in our study all sequences originate from ticks collected in eastern regions, there are approximately 3% nt differences and all clustered separately (see Fig 3). However, since the sequences originate from pools of ticks, they represent a consensus with the most represented nt at each position.

ALSV has previously been reported in China, Finland, France, Russia, and Germany [12, 13, 17, 19]. Low host specificity may contribute to its widespread distribution e.g., ALSV antibodies or ALSV RNA have been detected in goats and sheep from China, in a red deer from Germany and in numerous human serum samples from China [13, 17, 18, 91, 117]. Several different arthropods have been suggested as possible vectors, including ticks, especially *I*. *persulcatus*, *I*. *ricinus*, *Dermacentor reticulatus*, and mosquitoes [13, 17, 18, 118]. Furthermore, the JMVG, to which ALSV belongs, is divided into two phylogenetic clades, one including tick- and vertebrate-associated Jingemnviruses sequenced from human, monkey, cattle, bat, rodent, and tortoise samples. The second clade includes Jingmenviruses from insects, plants and fungi, suggesting their ubiquitous endemic distribution [19]. Recently, viruses have been isolated from white-footed mice in North America that have the highest sequence similarity to the NS3 and NS5 proteins of JMVG viruses and share <70% aa identity with ALSV. However, in contrast to Eurasian studies, no JMVG viruses have been detected in ticks or mosquitoes in North America, suggesting that other arthropod vectors may play a role in transmission or that there is no vector competence [118].

Given the high pool positivity of ALSV in Swiss ticks, its potential transmission to and pathogenicity in humans should be investigated. The high abundance of ALSV in ticks suggests also a reservoir in wild- and/or domestic animal species that remains to be identified. However, antibodies that recognize specific ALSV proteins or serological assays to screen patient sera and thereby evaluate the public health relevance of the virus are not available, at least not commercially. Moreover, besides information derived from computational analysis of the ALSV genome sequence, not much is known about the live cycle of the virus in mammalian and arthropod cells, or the products of RNA and protein synthesis.

In summary, this study provides a broad overview of viruses and selected non-viral pathogens in Swiss ticks. For example, we were able to show the significantly higher risk of finding ticks with ALSV in the cantons of Grisons and Schaffhausen and with TBEV in the cantons of St. Gallen and Zurich. We also show that in some regions several pathogens can occur simultaneously in the tick populations and that urban areas should not be neglected as potential risk areas. In this context, a recent retrospective serological study showed a high seroprevalence of IgG antibodies to spotted fever group *Rickettsiae* in *B*. *burgdorferi* seropositive individuals [119]. While the two different pathogens may have been acquired separately, the simultaneous transmission of *Rickettsiae* and a second pathogen by a single tick bite is at least possible, given the high pool prevalence of *Rickettsiae* found in our study. The high pool prevalence for *Rickettsia* and *Ehrlichia* of engorged ticks (approx. 55%) collected from wild animals may suggest a possible role of these species as reservoirs.

Finally, NGS facilitated the identification of viruses not previously detected in the area, ALSV in particular. Further studies to understand the biology of ALSV are imperative to assess its potential risk as emerging pathogen in humans and animals.

## Supporting information

S1 Table Geographical coordinates of the collection sites. (DOCX)

S2 Table Pool positivity (%) for specific non-viral and viral pathogens per canton and collection time point. (DOCX)

S3 Table Pool positivity (%) for non-viral pathogens in questing ticks of different gender/development stage. (DOCX)

S4 Table Viral and bacterial burden shown as p value in the tick pools according to different variables. (DOCX)

S5 Table Pool positivity (%) for TBEV and ALSV of the different gender/development stages of questing ticks. (DOCX)

## References

[1] De La Fuente J, Estrada-Pena A, Venzal JM, Kocan KM, Sonenshine DE. Overview: Ticks as vectors of pathogens that cause disease in humans and animals. Frontiers in Bioscience. 2008 May 113(18):6938–46. doi: 10.2741/3200 18508706

[2] Diuk-Wasser MA, Vanacker MC, Fernandez MP. Impact of Land Use Changes and Habitat Fragmentation on the Eco-epidemiology of Tick-Borne Diseases. J Med Entomol. 2021 Jul 1;58(4):1546–64. doi: 10.1093/jme/tjaa209 33095859

[3] Gritsun TS, Lashkevich VA, Gould EA. Tick-borne encephalitis. Antiviral Res. 2003;57(1–2):129–46. doi: 10.1016/s0166-3542(02)00206-1 12615309

[4] Ecker M, Allison SL, Meixner T, Heinz FX. Sequence analysis and genetic classification of tick-borne encephalitis viruses from Europe and Asia. Journal of General Virology. 1999 Jan 1;80(1):179–85. doi: 10.1099/0022-1317-80-1-179 9934700

[5] Grard G, Moureau G, Charrel RN, Lemasson JJ, Gonzalez JP, Gallian P, et al. Genetic characterization of tick-borne flaviviruses: New insights into evolution, pathogenetic determinants and taxonomy. Virology. 2007 Apr 25;361(1):80–92. doi: 10.1016/j.virol.2006.09.015 17169393

[6] Tonteri E, Kipar A, Voutilainen L, Vene S, Vaheri A, Vapalahti O, et al. The Three Subtypes of Tick-Borne Encephalitis Virus Induce Encephalitis in a Natural Host, the Bank Vole (Myodes glareolus). PLoS One. 2013 Dec 13;8(12):e81214. doi: 10.1371/journal.pone.0081214 24349041 PMC3862475

[7] Ott D, Ulrich K, Ginsbach P, Öhme R, Bock-Hensley O, Falk U, et al. Tick-borne encephalitis virus (TBEV) prevalence in field-collected ticks (Ixodes ricinus) and phylogenetic, structural and virulence analysis in a TBE high-risk endemic area in southwestern Germany. Parasit Vectors. 2020 Jun 11;13(1). 10.1186/s13071-020-04146-7 PMC7291635 32527288

[8] Dai X, Shang G, Lu S, Yang J, Xu J. A new subtype of eastern tick-borne encephalitis virus discovered in Qinghai-Tibet Plateau, China article. Emerg Microbes Infect. 2018 Dec 1;7(1). 10.1038/s41426-018-0081-6 PMC5915441 29691370

[9] Demina T V., Dzhioev YP, Verkhozina MM, Kozlova I V., Tkachev SE, Plyusnin A et al. Genotyping and characterization of the geographical distribution of tick-borne encephalitis virus variants with a set of molecular probes. J Med Virol. 2010 May 1;82(6):965–76. doi: 10.1002/jmv.21765 20419810

[10] Carpio KL, Thompson JK, Widen SG, Smith JK, Juelich TL, Clements DE, et al. Differences in Genetic Diversity of Mammalian Tick-Borne Flaviviruses. Viruses. 2023 Feb 1;15(2). doi: 10.3390/v15020281 36851495 PMC9959157

[11] Qin XC, Shi M, Tian JH, Lin XD, Gao DY, He JR, et al. A tick-borne segmented RNA virus contains genome segments derived from unsegmented viral ancestors. Proc Natl Acad Sci U S A. 2014 May 6;111(18):6744–9. doi: 10.1073/pnas.1324194111 24753611 PMC4020047

[12] Temmam S, Bigot T, Chrétien D, Gondard M, Pérot P, Pommelet V, et al. Insights into the Host Range, Genetic Diversity, and Geographical Distribution of Jingmenviruses. mSphere. 2019 Dec 18;4(6). doi: 10.1128/mSphere.00645-19 31694898 PMC6835211

[13] Khasnatinov M, Leonie Ebert C, Söder L, Kubinski M, Glanz J, Gregersen E, et al. Detection and Characterization of Alongshan Virus in Ticks and Tick Saliva from Lower Saxony, Germany with Serological Evidence for Viral Transmission to Game and Domestic Animals. Microorganisms. 2023 Feb 21;11(3):543. doi: 10.3390/microorganisms11030543 36985117 PMC10055853

[14] Wang ZD, Wang W, Wang NN, Qiu K, Zhang X, Tana G, et al. Prevalence of the emerging novel Alongshan virus infection in sheep and cattle in Inner Mongolia, northeastern China. Parasit Vectors. 2019 Sep 12;12(1):1–7. 31511049 10.1186/s13071-019-3707-1 PMC6740026

[15] Ladner JT, Wiley MR, Beitzel B, Auguste AJ, Dupuis AP, Lindquist ME, et al. A Multicomponent Animal Virus Isolated from Mosquitoes. Cell Host Microbe. 2016 Sep 14;20(3):357–67. doi: 10.1016/j.chom.2016.07.011 27569558 PMC5025392

[16] Villa EC, Maruyama SR, de Miranda-Santos IKF, Palacios G, Ladner JT. Complete Coding Genome Sequence for Mogiana Tick Virus, a Jingmenvirus Isolated from Ticks in Brazil. Genome Announc. 2017;5(18). 10.1128/genomeA.00232-17 PMC5477184 28473376

[17] Wang ZD, Wang B, Wei F, Han SZ, Zhang L, Yang ZT, et al. A New Segmented Virus Associated with Human Febrile Illness in China. New England Journal of Medicine. 2019 May 30;380(22):2116–25. doi: 10.1056/NEJMoa1805068 31141633

[18] Kuivanen S, Levanov L, Kareinen L, Sironen T, Jääskeläinen AJ, Plyusnin I, et al. Detection of novel tick-borne pathogen, Alongshan virus, in Ixodes ricinus ticks, south-eastern Finland, 2019. Euro Surveill. 2019 Jul 4;24(27). doi: 10.2807/1560-7917.ES.2019.24.27.1900394 31290392 PMC6628756

[19] Kholodilov IS, Litov AG, Klimentov AS, Belova OA, Polienko AE, Nikitin NA, et al. Isolation and Characterisation of Alongshan Virus in Russia. Viruses 2020, Vol 12, Page 362. 2020 Mar 26;12(4):362. doi: 10.3390/v12040362 32224888 PMC7232203

[20] Colmant AMG, Charrel RN, Coutard B. Jingmenviruses: Ubiquitous, understudied, segmented flavi-like viruses. Front Microbiol. 2022 Oct 10;13:4023. doi: 10.3389/fmicb.2022.997058 36299728 PMC9589506

[21] O’Connell S, Granström M, Gray JS, Stanek G. Epidemiology of European Lyme borreliosis. Zentralblatt fur Bakteriologie. 1998;287(3):229–40. doi: 10.1016/s0934-8840(98)80124-2 9563197

[22] Sykes RA, Makiello P. An estimate of Lyme borreliosis incidence in Western Europe †. J Public Health (Oxf). 2017 Mar 1;39(1):74–81. doi: 10.1093/pubmed/fdw017 26966194

[23] Müllegger R, Müllegger R. Infektionen: Lyme-Borreliose, Leptospirose und Rückfallfieber. Braun-Falco’s Dermatologie, Venerologie und Allergologie. Springer Reference Medizin. Springer, Berlin, Heidelberg. 2017.

[24] Zeckenübertragene Krankheiten–Lagebericht Schweiz [Internet]. [cited 2023 May 13]. Available from: https://www.bag.admin.ch/bag/de/home/krankheiten/ausbrueche-epidemien-pandemien/aktuelle-ausbrueche-epidemien/zeckenuebertragene-krankheiten.html

[25] Mora F, Cadenas N, Rais O, Jouda F, Douet V, Humair P françois, et al. Phenology of Ixodes ricinus and Infection with Borrelia burgdorferi sensu lato Along a North- and South-Facing Altitudinal Gradient on Chaumont Mountain, Switzerland. J Med Entomol. 2007 Jul 1;44(4):683–93. doi: 10.1603/0022-2585(2007)44[683:poirai]2.0.co;2 17695026

[26] Mora nmora´ F, Cadenas M, Rais O, Humair PFO, Douet VR, Moret J, et al. Identification of Host Bloodmeal Source and Borrelia burgdorferi Sensu Lato in -Collected Ixodes ricinus Ticks in Chaumont (Switzerland). J Med Entomol. 2007 Nov 1;44(6):1109–17. 18047213 10.1603/0022-2585(2007)44[1109:iohbsa]2.0.co;2

[27] Wicki R, Sauter P, Mettler C, Natsch A, Enzler T, Pusterla N, et al. Swiss Army Survey in Switzerland to Determine the Prevalence of Francisella tularensis, Members of the Ehrlichia phagocytophila Genogroup, Borrelia burgdorferi Sensu Lato, and Tick-Borne Encephalitis Virus in Ticks. Eur J Clin Microbiol Infect Dis. 2000 Jun;19(6):427–32. doi: 10.1007/s100960000283 10947217

[28] Blanco JR, Oteo JA. Rickettsiosis in Europe. Ann N Y Acad Sci. 2006 Oct 1;1078(1):26–33. doi: 10.1196/annals.1374.003 17114677

[29] Parola P, Paddock CD, Socolovschi C, Labruna MB, Mediannikov O, Kernif T, et al. Update on tick-borne rickettsioses around the world: A geographic approach. Clin Microbiol Rev. 2013 Oct;26(4):657–702. doi: 10.1128/CMR.00032-13 24092850 PMC3811236

[30] Rizzoli A, Silaghi C, Obiegala A, Rudolf I, Hubálek Z, Földvári G, et al. Ixodes ricinus and Its Transmitted Pathogens in Urban and Peri-Urban Areas in Europe: New Hazards and Relevance for Public Health. Front Public Health. 2014 Dec 1; 2:251. doi: 10.3389/fpubh.2014.00251 25520947 PMC4248671

[31] Oechslin CP, Heutschi D, Lenz N, Tischhauser W, Péter O, Rais O, et al. Prevalence of tick-borne pathogens in questing Ixodes ricinus ticks in urban and suburban areas of Switzerland. Parasit Vectors. 2017 Nov 9;10(1):1–18. 29121976 10.1186/s13071-017-2500-2 PMC5680829

[32] Boretti FS, Perreten A, Meli ML, Cattori V, Willi B, Wengi N, et al. Molecular Investigations of Rickettsia helvetica infection in dogs, foxes, humans, and Ixodes ticks. Appl Environ Microbiol. 2009;75(10):3230–7. doi: 10.1128/AEM.00220-09 19329665 PMC2681666

[33] Lommano E, Bertaiola L, Dupasquier C, Gern L. Infections and Coinfections of Questing Ixodes ricinus Ticks by Emerging Zoonotic Pathogens in Western Switzerland. Appl Environ Microbiol. 2012 Jul;78(13):4606–12. doi: 10.1128/AEM.07961-11 22522688 PMC3370488

[34] Beati L, Humair PF, Aeschlimann A, Raoult D. Identification of Spotted Fever Group Rickettsiae Isolated from Dermacentor Marginatus and Ixodes Ricinus Ticks Collected in Switzerland. Am J Trop Med Hyg. 1994 Aug 1;51(2):138–48. doi: 10.4269/ajtmh.1994.51.138 7915498

[35] Faccini-Martínez ÁA, García-Álvarez L, Hidalgo M, Oteo JA. Syndromic classification of rickettsioses: an approach for clinical practice. International Journal of Infectious Diseases. 2014 Nov 1;28:126–39. doi: 10.1016/j.ijid.2014.05.025 25242696

[36] Madison-Antenucci S, Kramer LD, Gebhardt LL, Kauffman E. Emerging Tick-Borne Diseases. Clin Microbiol Rev. 2020 Jan 2;33(2):e00083–18. doi: 10.1128/CMR.00083-18 31896541 PMC6941843

[37] Wass L, Grankvist A, Bell-Sakyi L, Bergström M, Ulfhammer E, Lingblom C, et al. Cultivation of the causative agent of human neoehrlichiosis from clinical isolates identifies vascular endothelium as a target of infection. Emerg Microbes Infect. 2019;8(1):413–425. doi: 10.1080/22221751.2019.1584017 30898074 PMC6455172

[38] Portillo A, Santibáñez P, Palomar AM, Santibáñez S, Oteo JA. New Microbes New Infect. 2018 Mar 22:6. 29556406 10.1016/j.nmni.2017.12.011 PMC5857181

[39] Kawahara M, Rikihisa Y, Isogai E, Takahashi M, Misumi H, Suto C, et al. Ultrastructure and phylogenetic analysis of “Candidatus Neoehrlichia mikurensis” in the family Anaplasmataceae, isolated from wild rats and found in Ixodes ovatus ticks. Int J Syst Evol Microbiol. 2004 Sep 1;54(5):1837–43. doi: 10.1099/ijs.0.63260-0 15388752

[40] Silaghi C, Beck R, Oteo JA, Pfeffer M, Sprong H. Neoehrlichiosis: an emerging tick-borne zoonosis caused by Candidatus Neoehrlichia mikurensis. Exp Appl Acarol. 2016 Mar 1;68(3):279–97. doi: 10.1007/s10493-015-9935-y 26081117

[41] Fehr JS, Bloemberg G V., Ritter C, Hombach M, Lüscher TF, Weber R, et al. v Emerg Infect Dis. 2010;16(7):1127. 20587186 10.3201/eid1607.091907 PMC3358111

[42] Ondruš J, Kulich P, Sychra O, Široký P. Putative morphology of Neoehrlichia mikurensis in salivary glands of Ixodes ricinus. Scientific Reports 2020 10:1. 2020 Sep 29;10(1):1–5. doi: 10.1038/s41598-020-72953-0 32994495 PMC7525475

[43] Telford SR 3rd, Goethert HK. Ecology of Francisella tularensis. Annu Rev Entomol. 2020;65:351–372. doi: 10.1146/annurev-ento-011019-025134 31600457 PMC8300880

[44] Johansson A, Celli J, Conlan W, Elkins KL, Forsman M, Keim PS, et al. Objections to the transfer of Francisella novicida to the subspecies rank of Francisella tularensis. Int J Syst Evol Microbiol. 2010 Aug 1;60(8):1717–8. 20688748 10.1099/ijs.0.022830-0 PMC7442299

[45] Timofeev V, Bakhteeva I, Titareva G, Kopylov P, Christiany D, Mokrievich A, et al. Russian isolates enlarge the known geographic diversity of Francisella tularensis subsp. mediasiatica. PLoS One. 2017 Sep 1;12(9). 10.1371/journal.pone.0183714 PMC5584958 28873421

[46] Tularaemia—Annual Epidemiological Report for 2019 [Internet]. [cited 2023 Feb 18]. Available from: https://www.ecdc.europa.eu/en/publications-data/tularaemia-annual-epidemiological-report-2019

[47] Selbitz HJ, Truyen U, Valentin-Weigand P, Alber G, Amtsberg G, Bauer J, et al. Tiermedizinische Mikrobiologie, Infektions- und Seuchenlehre. Tiermedizinische Mikrobiologie, Infektions- und Seuchenlehre. Georg Thieme Verlag KG, Stuttgart; 2011.

[48] Meldepflichtige Infektionskrankheiten–Wöchentliche Fallzahlen [Internet]. [cited 2023 May 25]. Available from: https://www.bag.admin.ch/bag/de/home/zahlen-und-statistiken/zahlen-zu-infektionskrankheiten/meldepflichtige-infektionskrankheiten—woechentliche-fallzahlen.html

[49] Bajer A, Beck A, Beck R, Behnke JM, Dwużnik-Szarek D, Eichenberger RM, et al. Babesiosis in Southeastern, Central and Northeastern Europe: An Emerging and Re-Emerging Tick-Borne Disease of Humans and Animals. Microorganisms. 2022 May 1;10(5):945. doi: 10.3390/microorganisms10050945 35630388 PMC9146636

[50] Usatii N, Khachatrian A, Stratidis J. Spontaneous splenic rupture due to Babesia microti infection: Case report and review of the literature. IDCases. 2014 Jan 1;1(4):63–5. doi: 10.1016/j.idcr.2014.08.002 26839774 PMC4735021

[51] Rosner F, Zarrabi MH, Benach JL, Habicht GS. Babesiosis in splenectomized adults: Review of 22 reported cases. Am J Med. 1984 Apr 1;76(4):696–701. 6424470 10.1016/0002-9343(84)90298-5

[52] Vannier EG, Diuk-Wasser MA, Ben Mamoun C, Krause PJ. Babesiosis. Infect Dis Clin North Am. 2015 Jun 1;29(2):357. doi: 10.1016/j.idc.2015.02.008 25999229 PMC4458703

[53] Schorn S, Pfister K, Reulen H, Mahling M, Silaghi C. Occurrence of Babesia spp., Rickettsia spp. and Bartonella spp. in Ixodes ricinus in Bavarian public parks, Germany. Parasit Vectors. 2011 Jul 15;4(1):1–9. doi: 10.1186/1756-3305-4-135 21762494 PMC3154157

[54] Michelet L, Delannoy S, Devillers E, Umhang G, Aspan A, Juremalm M, et al. High-throughput screening of tick-borne pathogens in Europe. Front Cell Infect Microbiol. 2014;4:103. doi: 10.3389/fcimb.2014.00103 25120960 PMC4114295

[55] Øines Ø, Radzijevskaja J, Paulauskas A, Rosef O. Prevalence and diversity of Babesia spp. in questing Ixodes ricinus ticks from Norway. Parasit Vectors. 2012 Aug 4;5(1):1–8. 22862883 10.1186/1756-3305-5-156 PMC3439691

[56] Estrada-Peña A. Ticks of Domestic Animals in the Mediterranean Region. A Guide to Identification of Species. University of Zaragoza; 2004.

[57] Halos L, Jamal T, Vial L, Maillard R, Suau A, Le Menach A, et al. Determination of an efficient and reliable method for DNA extraction from ticks. Vet Res. 2004;35:709–13. doi: 10.1051/vetres:2004038 15535960

[58] Maurer FP, Keller PM, Beuret C, Joha C, Achermann Y, Gubler J, et al. Close geographic association of human neoehrlichiosis and tick populations carrying “Candidatus Neoehrlichia mikurensis” in eastern Switzerland. J Clin Microbiol. 2013 Jan;51(1):169–76. doi: 10.1128/JCM.01955-12 23115262 PMC3536216

[59] Kubacki J, Flacio E, Qi W, Guidi V, Tonolla M, Fraefel C. Viral Metagenomic Analysis of Aedes albopictus Mosquitos from Southern Switzerland. Viruses. 2020 Aug 24;12(9):929. doi: 10.3390/v12090929 32846980 PMC7552062

[60] Kubacki J, Qi W, Fraefel C. Differential Viral Genome Diversity of Healthy and RSS-Affected Broiler Flocks. Microorganisms. 2022 May 25;10(6):1092. doi: 10.3390/microorganisms10061092 35744610 PMC9231120

[61] Hardmeier I, Aeberhard N, Qi W, Schoenbaechler K, Kraettli H, Hatt JM, et al. Metagenomic analysis of fecal and tissue samples from 18 endemic bat species in Switzerland revealed a diverse virus composition including potentially zoonotic viruses. PLoS One. 2021 Jun 1;16(6):e0252534. doi: 10.1371/journal.pone.0252534 34133435 PMC8208571

[62] Nurk S, Meleshko D, Korobeynikov A, Pevzner PA. MetaSPAdes: A new versatile metagenomic assembler. Genome Res. 2017 May 1;27(5):824–34. doi: 10.1101/gr.213959.116 28298430 PMC5411777

[63] Tamura K, Stecher G, Kumar S. MEGA11: Molecular Evolutionary Genetics Analysis Version 11. Mol Biol Evol. 2021 Jun 25;38(7):3022–7. doi: 10.1093/molbev/msab120 33892491 PMC8233496

[64] R Core Team. R: A language and environment for statistical computing. R Foundation for Statistical Computing, Vienna, Austria. URL https://www.R-project.org/.

[65] Hasan M.M. and Dunn P.K. Understanding the effect of climatology on monthly rainfall amounts in Australia using Tweedie GLMs. International Journal of Climatology, 2012;32(7) 1006–1017.

[66] Giner G, Smyth GK. statmod: Probability Calculations for the Inverse Gaussian Distribution. R Journal. 2016;8(1), 339–351.

[67] Pettersson JHO, Shi M, Bohlin J, Eldholm V, Brynildsrud OB, Paulsen KM, et al. Characterizing the virome of Ixodes ricinus ticks from northern Europe. Sci Rep. 2017 Dec 1;7(1). doi: 10.1038/s41598-017-11439-y 28883464 PMC5589870

[68] Vanmechelen B, Laenen L, Vergote V, Maes P. Grotenhout Virus, a Novel Nairovirus Found in Ixodes ricinus in Belgium. Genome Announc. 2017;5(21). 10.1128/genomeA.00288-17 PMC5477388 28546475

[69] Daveu R, Hervet C, Sigrist L, Sassera D, Jex A, Labadie K, et al. Sequence diversity and evolution of a group of iflaviruses associated with ticks. Arch Virol. 2021 Jul 1;166(7):1843. doi: 10.1007/s00705-021-05060-8 33870470 PMC8195936

[70] Stegmüller S, Fraefel C, Kubacki J. Genome Sequence of Alongshan Virus from Ixodes ricinus Ticks Collected in Switzerland. Microbiol Resour Announc. 2023 Mar 16;12(3). doi: 10.1128/mra.01287-22 36779723 PMC10019301

[71] Sprong H, Wielinga PR, Fonville M, Reusken C, Brandenburg AH, Borgsteede F, et al. Ixodes ricinus ticks are reservoir hosts for Rickettsia helvetica and potentially carry flea-borne Rickettsia species. Parasit Vectors. 2009 Sep 4;2(1):1–7. doi: 10.1186/1756-3305-2-41 19732416 PMC2743653

[72] Guccione C, Colomba C, Iaria C, Cascio A. Rickettsiales in the WHO European Region: an update from a One Health perspective. Parasit Vectors. 2023 Dec 1;16(1):1–15. 36717936 10.1186/s13071-022-05646-4 PMC9885594

[73] Baumann D, Pusterla N, Péter O, Grimm F, Fournier PE, Schär G, et al. Fever after a tick bite: clinical manifestations and diagnosis of acute tick bite-associated infections in northeastern Switzerland. Dtsch Med Wochenschr. 2003 May 1;128(19):1042–7. 12736854 10.1055/s-2003-39103

[74] Blanco JR, Oteo JA. Human granulocytic ehrlichiosis in Europe. Clinical Microbiology and Infection. 2002 Dec 1;8(12):763–72. doi: 10.1046/j.1469-0691.2002.00557.x 12519349

[75] Human granulocytic anaplasmosis [Internet]. [cited 2023 May 29]. Available from: https://www.ecdc.europa.eu/en/human-granulocytic-anaplasmosis

[76] Stuen S. Anaplasma Phagocytophilum—The most widespread tick-borne infection in animals in Europe. Vet Res Commun. 2007 Aug 16;31(SUPPL. 1):79–84. 17682851 10.1007/s11259-007-0071-y

[77] Hofmann-Lehmann R, Wagmann N, Meli ML, Riond B, Novacco M, Joekel D, et al. Detection of “Candidatus Neoehrlichia mikurensis” and other Anaplasmataceae and Rickettsiaceae in Canidae in Switzerland and Mediterranean countries. Schweiz Arch Tierheilkd. 2016 Oct 1;158(10):691–700. 27707682 10.17236/sat00087

[78] Pusterla N, Huder JB, Leutenegger CM, Braun U, Madigan JE, Lutz H. Quantitative Real-Time PCR for Detection of Members of the Ehrlichia phagocytophila Genogroup in Host Animals and Ixodes ricinus Ticks. J Clin Microbiol. 1999;37(5):1329–31. doi: 10.1128/JCM.37.5.1329-1331.1999 10203480 PMC84766

[79] Ebani VV, Verin R, Fratini F, Poli A, Cerri D. Molecular Survey of Anaplasma phagocytophilum and Ehrlichia canis in Red Foxes (Vulpes vulpes) from Central Italy. J Wildl Dis. 2011;47(3):699–703. doi: 10.7589/0090-3558-47.3.699 21719836

[80] Sándor SH, Nóra AB, Alexandra T, Jen J, Dorottya K, Balázs F, et al. Anaplasmataceae closely related to Ehrlichia chaffeensis and Neorickettsia helminthoeca from birds in Central Europe, Hungary. Antonie Van Leeuwenhoek. 2020 Jul;113(7):1067–1073. doi: 10.1007/s10482-020-01415-4 32318980 PMC7272389

[81] Oteo JA, Brouqui P. Ehrlichiosis and human anaplasmosis. Enferm Infecc Microbiol Clin. 2005 Jun 1;23(6):375–80. 15970171 10.1157/13076178

[82] Goel R, Westblade LF, Kessler DA, Sfeir M, Slavinski S, Backenson B, et al. Death from Transfusion-Transmitted Anaplasmosis. Emerg Infect Dis. 2018 Aug 1;24(8):1548–50. 30016241 10.3201/eid2408.172048 PMC6056119

[83] Burri C, Dupasquier C, Bastic V, Gern L. Pathogens of Emerging Tick-Borne Diseases, Anaplasma phagocytophilum, Rickettsia spp., and Babesia spp., in Ixodes Ticks Collected from Rodents at Four Sites in Switzerland (Canton of Bern). Vector Borne Zoonotic Dis. 2011 Jul;11(7):939–44. doi: 10.1089/vbz.2010.0215 21417929

[84] Steinbrink A, Brugger K, Margos G, Kraiczy P, Klimpel S. The evolving story of Borrelia burgdorferi sensu lato transmission in Europe. Parasitology Research. 2022 Feb 5;121(3):781–803. doi: 10.1007/s00436-022-07445-3 35122516 PMC8816687

[85] Hansford KM, Wheeler BW, Tschirren B, Medlock JM. Questing Ixodes ricinus ticks and Borrelia spp. in urban green space across Europe: A review. Zoonoses Public Health. 2022 May 1;69(3):153–66. 35122422 10.1111/zph.12913 PMC9487987

[86] Anderson JF, Johnson RC, Magnarelli LA. Seasonal prevalence of Borrelia burgdorferi in natural populations of white-footed mice, Peromyscus leucopus. J Clin Microbiol. 1987 Aug;25(8):1564–6. doi: 10.1128/jcm.25.8.1564-1566.1987 3624451 PMC269274

[87] Donahue JG, Piesman J, Spielman A. Reservoir competence of white-footed mice for Lyme disease spirochetes. Am J Trop Med Hyg. 1987 Jan;36(1):92–6. doi: 10.4269/ajtmh.1987.36.92 3812887

[88] Bunikis J, Tsao J, Luke CJ, Luna MG, Fish D, Barbour AG. Borrelia burgdorferi infection in a natural population of Peromyscus Leucopus mice: a longitudinal study in an area where Lyme Borreliosis is highly endemic. J Infect Dis. 2004 Apr 15;189(8):1515–23. doi: 10.1086/382594 15073690

[89] Gigandet L, Stauffer E, Douet V, Rais O, Moret J, Gern L. Prevalence of Three Zoonotic Babesia Species in Ixodes ricinus (Linné, 1758) Nymphs in a Suburban Forest in Switzerland. Vector Borne Zoonotic Dis. 2011 Apr;11(4):363–6. 21395425 10.1089/vbz.2010.0195

[90] Blaschitz M, Narodoslavsky-Gföller M, Kanzler M, Stanek G, Walochnik J. Babesia species occurring in Austrian Ixodes ricinus ticks. Appl Environ Microbiol. 2008 Aug;74(15):4841–6. doi: 10.1128/AEM.00035-08 18539787 PMC2519353

[91] Wittwer M, Altpeter E, Pilo P, Gygli SM, Beuret C, Foucault F, et al. Population Genomics of Francisella tularensis subsp. holarctica and its Implication on the eco-epidemiology of tularemia in Switzerland. Front Cell Infect Microbiol. 2018 Mar 22;8: 89. doi: 10.3389/fcimb.2018.00089 29623260 PMC5875085

[92] Schütz SD, Liechti N, Altpeter E, Labutin A, Wütrich T, Schmidt KM, et al. Phylogeography of Francisella tularensis subspecies holarctica and epidemiology of tularemia in Switzerland. Front Microbiol. 2023;14:1151049. doi: 10.3389/fmicb.2023.1151049 37113234 PMC10126411

[93] Vandegrift KJ, Kapoor A. The Ecology of New Constituents of the Tick Virome and Their Relevance to Public Health. Viruses. 2019 Jun 7;11(6):529. doi: 10.3390/v11060529 31181599 PMC6630940

[94] Cai X, Cai X, Xu Y, Shao Y, Fu L, Men X, et al. Virome analysis of ticks and tick-borne viruses in Heilongjiang and Jilin Provinces, China. Virus Res. 2023 Jan 2;323:199006. doi: 10.1016/j.virusres.2022.199006 36414189 PMC10194156

[95] Harvey E, Rose K, Edena JS, Lo N, Abeyasuriya T, Shi M, et al. Extensive Diversity of RNA Viruses in Australian Ticks. J Virol. 2019 Jan 17;93(3):e01358–18. doi: 10.1128/JVI.01358-18 30404810 PMC6340049

[96] Moutailler S, Popovici I, Devillers E, Vayssier-Taussat M, Eloit M. Diversity of viruses in Ixodes ricinus, and characterization of a neurotropic strain of Eyach virus. New Microbes New Infect. 2016 May 1;11:71–81. doi: 10.1016/j.nmni.2016.02.012 27158509 PMC4845080

[97] Kong Y, Zhang G, Jiang L, Wang P, Zhang S, Zheng X, et al. Metatranscriptomics Reveals the Diversity of the Tick Virome in Northwest China. Microbiol Spectr. 2022 Oct 26;10(5). doi: 10.1128/spectrum.01115-22 36214702 PMC9602664

[98] Garrison AR, Alkhovsky S V., Avšič-Županc T, Bente DA Bergeron É, Burt F, et al. ICTV virus taxonomy profile: Nairoviridae. Journal of General Virology. 2020;101(8):798–9. doi: 10.1099/jgv.0.001485 32840475 PMC7641396

[99] Ni XB, Cui XM, Liu JY, Ye RZ, Wu YQ, Jiang JF, et al. Metavirome of 31 tick species provides a compendium of 1,801 RNA virus genomes. Nature Microbiology. 2023 Jan 5;8(1):162–73. doi: 10.1038/s41564-022-01275-w 36604510 PMC9816062

[100] Daoudi M, Romeo G, Marzani K, Petrella A, Bonilauri P, Lelli D, et al. New Isolation of Ponticelli III Virus (Bunyavirales: Phenuiviridae) in Emilia-Romagna Region, Italy. Viruses. 2023 Feb 1;15(2):422. doi: 10.3390/v15020422 36851636 PMC9964127

[101] Vanmechelen B, Merino M, Vergote V, Laenen L, Thijssen M, Martí-Carreras J, et al. Exploration of the Ixodes ricinus virosphere unveils an extensive virus diversity including novel coltiviruses and other reoviruses. Virus Evol. 2021;7(2). doi: 10.1093/ve/veab066 34532065 PMC8438917

[102] Xu L, Guo M, Hu B, Zhou H, Yang W, Hui L, et al. Tick virome diversity in Hubei Province, China, and the influence of host ecology. Virus Evol. 2021 Dec 16;7(2):1–12. 10.1093/ve/veab089 PMC8599308 34804590

[103] Valles SM, Chen Y, Firth AE, Guérin DMA, Hashimoto Y, Herrero S, et al. ICTV virus taxonomy profile: Iflaviridae. Journal of General Virology. 2017 Apr 1;98(4):527–8. doi: 10.1099/jgv.0.000757 28382900 PMC5657024

[104] Nakao R, Matsuno K, Qiu Y, Maruyama J, Eguchi N, Nao N, et al. Putative RNA viral sequences detected in an Ixodes scapularis-derived cell line. Ticks Tick Borne Dis. 2017 Jan 1;8(1):103–11. doi: 10.1016/j.ttbdis.2016.10.005 27769656

[105] Silva LA, Ardisson-Araujo DMP, Tinoco RS, Fernandes OA, Melo FL, Ribeiro BM. Complete genome sequence and structural characterization of a novel iflavirus isolated from Opsiphanes invirae (Lepidoptera: Nymphalidae). J Invertebr Pathol. 2015 Sep 1;130:136–40. doi: 10.1016/j.jip.2015.08.001 26254043

[106] Zhao T, Gong H, Shen X, Zhang W, Shan T, Yu X, et al. Comparison of Viromes in Ticks from Different Domestic Animals in China. Virol Sin. 2020;35(4):398–406. doi: 10.1007/s12250-020-00197-3 32157603 PMC7462941

[107] Frühsommer-Meningoenzephalitis (FSME) [Internet]. [cited 2023 May 26]. Available from: https://www.bag.admin.ch/bag/de/home/krankheiten/krankheiten-im-ueberblick/fsme.html

[108] European Centre for Disease Prevention and Control. Tick-borne encephalitis. In: ECDC. Annual epidemiological report for 2020. Stockholm: ECDC; 2022.

[109] Süss J. Tick-borne encephalitis 2010: epidemiology, risk areas, and virus strains in Europe and Asia-an overview. Ticks Tick Borne Dis. 2011 Mar;2(1):2–15. doi: 10.1016/j.ttbdis.2010.10.007 21771531

[110] Soleng A, Edgar KS, Paulsen KM, Pedersen BN, Okbaldet YB, Skjetne IEB, et al. Distribution of Ixodes ricinus ticks and prevalence of tick-borne encephalitis virus among questing ticks in the Arctic Circle region of northern Norway. Ticks Tick Borne Dis. 2018 Jan 1;9(1):97–103. doi: 10.1016/j.ttbdis.2017.10.002 29030314

[111] Wójcik-Fatla A, Cisak E, Zajac V, Zwoliński J, Dutkiewicz J. Prevalence of tick-borne encephalitis virus in Ixodes ricinus and Dermacentor reticulatus ticks collected from the Lublin region (eastern Poland). Ticks Tick Borne Dis. 2011 Mar;2(1):16–9. doi: 10.1016/j.ttbdis.2010.10.001 21771532

[112] Bestehorn M, Weigold S, Kern W V., Chitimia-Dobler L, MacKenstedt U, Dobler G et al. Phylogenetics of tick-borne encephalitis virus in endemic foci in the upper Rhine region in France and Germany. PLoS One. 2018 Oct 1;13(10):e0204790. doi: 10.1371/journal.pone.0204790 30335778 PMC6193627

[113] Carpi G, Bertolotti L, Rosati S, Rizzoli A. Prevalence and genetic variability of tick-borne encephalitis virus in host-seeking Ixodes ricinus in northern Italy. Journal of General Virology. 2009 Dec 1;90(12):2877–83. doi: 10.1099/vir.0.013367-0 19675189

[114] Rieille N, Bressanelli S, Freire CCM, Arcioni S, Gern L, Péter O, et al. Prevalence and phylogenetic analysis of tick-borne encephalitis virus (TBEV) in field-collected ticks (Ixodes ricinus) in southern Switzerland. Parasit Vectors. 2014 Sep 22;7(1):1–13. 25245773 10.1186/1756-3305-7-443 PMC4261884

[115] Lommano E, Burri C, Maeder G, Guerne M, Bastic V, Patalas E, et al. Prevalence and Genotyping of Tick-Borne Encephalitis Virus in Questing Ixodes ricinus Ticks in a New Endemic Area in Western Switzerland. J Med Entomol. 2012 Jan 1;49(1):156–64. doi: 10.1603/me11044 22308784

[116] Michelitsch A, Wernike K, Klaus C, Dobler G, Beer M. Exploring the Reservoir Hosts of Tick-Borne Encephalitis Virus. Viruses. 2019 Jul 1;11(7). doi: 10.3390/v11070669 31336624 PMC6669706

[117] Kholodilov IS, Belova OA, Morozkin ES, Litov AG, Ivannikova AY, Makenov MT, et al. Geographical and Tick-Dependent Distribution of Flavi-Like Alongshan and Yanggou Tick Viruses in Russia. Viruses. 2021 Mar 11;13(3):458. doi: 10.3390/v13030458 33799742 PMC7998622

[118] Vandegrift KJ, Kumar A, Sharma H, Murthy S, Kramer LD, Ostfeld R, et al. Presence of segmented flavivirus infections in North America. Emerg Infect Dis. 2020 Aug 1;26(8):1810–7. doi: 10.3201/eid2608.190986 32687041 PMC7392405

[119] Kosak L, Satz N, Jutzi M, Dobec M, Schlagenhauf P. Spotted fever group rickettsiae and Anaplasma phagocytophilum in Borrelia burgdorferi sensu lato seropositive individuals with or without Lyme disease: A retrospective analysis. New Microbes New Infect. 2023 Jun 1;53:101139. doi: 10.1016/j.nmni.2023.101139 37168237 PMC10165448

