# Supplementary material for: Hazard potential of Swiss Ixodes ricinus ticks: Virome composition and presence of selected bacterial and protozoan pathogens
Source: PLoS One. 2023 Nov 13;18(11):e0290942. doi: 10.1371/journal.pone.0290942 (PMC10642849; doi:10.1371/journal.pone.0290942)
Supplement: S1 Table — (DOCX) [file pone.0290942.s001.docx]

Supporting information

**S1 Table. Geographical coordinates of the collection sites**

| **Canton** | **Environment** | **Latitude** | **Longitude** |
| --- | --- | --- | --- |
| **SO** | rural 1 | 47.397111N | 7.453278E |
|  | rural 2 | 47.39525N | 7.451472E |
|  | urban | 47.221N | 7.536028E |
| **BE** | rural | 47.009722N | 7.617222E |
|  | urban | 46.957056N | 7.422333E |
| **GE** | rural | 46.176028N | 6.019222E |
|  | urban | 46.210306N | 6.101194E |
| **VS** | rural | 46.192722N | 7.024778E |
|  | urban | 46.233333N | 7.340083E |
| **TI** | rural | 46.202278N | 8.68375E |
|  | urban | 46.182528N | 9.026917E |
| **GR** | rural | 46.794917N | 9.401556E |
|  | urban | 46.871111N | 9.54475E |
| **JU** | rural | 47.242417N | 6.954778E |
|  | urban | 47.3735N | 7.338722E |
| **SG** | rural 1 | 47.413056N | 9.419667E |
|  | rural 2 | 47.431222N | 9.451222E |
|  | urban | 47.41325N | 9.368194E |
| **SH** | rural | 47.673306N | 8.535833E |
|  | urban 1 | 47.693583N | 8.615417E |
|  | urban 2 | 47.715444N | 8.637944E |
| **ZH** | rural | 47.623028N | 8.694306E |
|  | urban 1 | 47.399111N | 8.554306E |
|  | urban 2 | 47.40175N | 8.519167E |
