# Supplementary material for: Hazard potential of Swiss Ixodes ricinus ticks: Virome composition and presence of selected bacterial and protozoan pathogens
Source: PLoS One. 2023 Nov 13;18(11):e0290942. doi: 10.1371/journal.pone.0290942 (PMC10642849; doi:10.1371/journal.pone.0290942)
Supplement: S2 Table — (DOCX) [file pone.0290942.s002.docx]

**S2 Table. Pool positivity (%) for specific non-viral and viral pathogens per canton and collection time point.**

| **Canton** | **Collection timepoint** | **Rickettsia sp.** | | **Ehrlichia sp.** | | **Borrelia sp.** | | **Neoehrlichia mikurensis.** | | **Babesia sp.** | | **Francisella tularensis** | | **TBEV** | | **ALSV** | | | |
| --- | --- | --- | --- | --- | --- | --- | --- | --- | --- | --- | --- | --- | --- | --- | --- | --- | --- | --- | --- |
|  |  | **R** | **U** | **R** | **U** | **R** | **U** | **R** | **U** | **R** | **U** | **R** | **U** | **R** | **U** | **R** | **U** |  |  |
| **SO** | May 2021 | 82 | 100 | 40 | 100 | 2 | 33 | 0 | 0 | 0 | 0 | 0 | 0 | 0 | 0 | 0 | 0 |  |  |
|  | Sept. 2021 | 33 | 100 | 33 | 100 | 0 | 0 | 0 | 0 | 0 | 0 | 0 | 0 | 0 | 0 | 0 | 0 |  |  |
|  | May 2022 | 90 | 50 | 27 | 50 | 0 | 0 | 0 | 0 | 0 | 0 | 0 | 0 | 0 | 0 | 0 | 0 |  |  |
|  | Sept. 2022 | 33 | 100 | 33 | 50 | 0 | 0 | 0 | 0 | 0 | 0 | 0 | 0 | 0 | 0 | 0 | 0 |  |  |
| **BE** | May 2021 | 90 | 80 | 10 | 80 | 0 | 0 | 0 | 20 | 0 | 0 | 0 | 0 | 0 | 0 | 0 | 0 |  |  |
|  | Sept. 2021 | 100 | 100 | 0 | 100 | 0 | 0 | 0 | 0 | 0 | 0 | 0 | 0 | 0 | 0 | 0 | 0 |  |  |
|  | May 2022 | 100 | 100 | 50 | 60 | 0 | 0 | 0 | 0 | 0 | 0 | 0 | 0 | 0 | 0 | 0 | 0 |  |  |
|  | Sept. 2022 | 50 | 0 | 50 | 0 | 0 | 50 | 0 | 0 | 0 | 0 | 0 | 50 | 0 | 0 | 0 | 0 |  |  |
| **GE** | May 2021 | 50 | 100 | 50 | 50 | 50 | 50 | 50 | 50 | 0 | 50 | 0 | 0 | 0 | 0 | 0 | 0 |  |  |
|  | Sept. 2021 | 0 | 0 | 0 | 0 | 0 | 0 | 0 | 0 | 0 | 0 | 0 | 0 | 0 | 0 | 0 | 0 |  |  |
|  | May 2022 | 0 | 100 | 0 | 0 | 0 | 0 | 0 | 0 | 0 | 0 | 0 | 0 | 0 | 0 | 0 | 0 |  |  |
|  | Sept. 2022 | 0 | 0 | 0 | 0 | 0 | 0 | 0 | 0 | 0 | 0 | 0 | 0 | 0 | 0 | 0 | 0 |  |  |
| **VS** | May 2021 | 33 | 100 | 33 | 0 | 0 | 100 | 33 | 0 | 0 | 0 | 0 | 0 | 0 | 0 | 0 | 0 |  |  |
|  | Sept. 2021 | 0 | 0 | 0 | 0 | 0 | 0 | 0 | 0 | 0 | 0 | 0 | 0 | 0 | 0 | 0 | 0 |  |  |
|  | May 2022 | 25 | 60 | 50 | 0 | 0 | 0 | 0 | 0 | 0 | 0 | 0 | 0 | 0 | 0 | 0 | 0 |  |  |
|  | Sept. 2022 | 0 | 100 | 50 | 0 | 50 | 0 | 0 | 0 | 0 | 0 | 0 | 0 | 0 | 0 | 0 | 0 |  |  |
| **TI** | May 2021 | 100 | 100 | 66 | 33 | 0 | 0 | 0 | 0 | 0 | 0 | 0 | 0 | 0 | 0 | 0 | 0 |  |  |
|  | Sept. 2021 | 0 | 0 | 0 | 0 | 0 | 0 | 0 | 0 | 0 | 0 | 0 | 0 | 0 | 0 | 0 | 0 |  |  |
|  | May 2022 | 100 | 100 | 75 | 25 | 0 | 0 | 0 | 0 | 0 | 0 | 0 | 0 | 0 | 0 | 0 | 0 |  |  |
|  | Sept. 2022 | 100 | 0 | 0 | 0 | 0 | 0 | 0 | 0 | 0 | 0 | 0 | 0 | 0 | 0 | 0 | 0 |  |  |
| **GR** | May 2021 | 50 | 60 | 50 | 80 | 0 | 0 | 0 | 0 | 0 | 0 | 0 | 0 | 12 | 0 | 12 | 0 |  |  |
|  | Sept. 2021 | 0 | 50 | 50 | 100 | 0 | 0 | 0 | 0 | 0 | 0 | 0 | 0 | 0 | 0 | 0 | 0 |  |  |
|  | May 2022 | 46 | 50 | 53 | 60 | 0 | 10 | 0 | 0 | 0 | 0 | 0 | 0 | 6 | 10 | 46 | 20 |  |  |
|  | Sept. 2022 | 66 | 50 | 66 | 50 | 0 | 33 | 0 | 0 | 0 | 0 | 0 | 0 | 0 | 66 | 33 | 0 |  |  |
| **JU** | May 2021 | 75 | 100 | 33 | 50 | 0 | 25 | 0 | 0 | 0 | 0 | 0 | 0 | 0 | 0 | 0 | 0 |  |  |
|  | Sept. 2021 | 33 | 0 | 33 | 50 | 0 | 0 | 0 | 50 | 0 | 0 | 0 | 0 | 0 | 0 | 0 | 0 |  |  |
|  | May 2022 | 58 | 50 | 0 | 25 | 0 | 0 | 0 | 0 | 0 | 0 | 0 | 0 | 0 | 0 | 0 | 0 |  |  |
|  | Sept. 2022 | 22 | 50 | 11 | 50 | 0 | 0 | 0 | 0 | 0 | 0 | 0 | 0 | 0 | 0 | 0 | 0 |  |  |
| **SG** | May 2021 | 33 | 100 | 33 | 0 | 0 | 0 | 0 | 0 | 0 | 0 | 0 | 0 | 8 | 0 | 0 | 0 |  |  |
|  | Sept. 2021 | 0 | 50 | 0 | 50 | 0 | 0 | 0 | 0 | 0 | 0 | 0 | 0 | 0 | 0 | 0 | 0 |  |  |
|  | May 2022 | 72 | 85 | 27 | 71 | 9 | 0 | 0 | 0 | 0 | 0 | 0 | 0 | 63 | 0 | 0 | 0 |  |  |
|  | Sept. 2022 | 100 | 100 | 0 | 66 | 0 | 33 | 0 | 0 | 0 | 0 | 0 | 0 | 0 | 0 | 0 | 0 |  |  |
| **SH** | May 2021 | 100 | 94 | 66 | 29 | 0 | 5 | 0 | 0 | 0 | 0 | 0 | 0 | 0 | 0 | 0 | 17 |  |  |
|  | Sept. 2021 | 0 | 75 | 0 | 25 | 0 | 0 | 0 | 0 | 0 | 0 | 0 | 0 | 0 | 0 | 0 | 50 |  |  |
|  | May 2022 | 66 | 100 | 33 | 38 | 0 | 4 | 0 | 0 | 0 | 0 | 0 | 0 | 0 | 0 | 33 | 42 |  |  |
|  | Sept. 2022 | 50 | 100 | 0 | 16 | 0 | 0 | 0 | 0 | 0 | 0 | 0 | 0 | 0 | 16 | 50 | 33 |  |  |
| **ZH** | May 2021 | 100 | 90 | 75 | 30 | 25 | 10 | 0 | 0 | 0 | 0 | 0 | 0 | 0 | 10 | 0 | 0 |  |  |
|  | Sept. 2021 | 100 | 50 | 100 | 50 | 0 | 0 | 100 | 0 | 0 | 0 | 0 | 0 | 0 | 0 | 0 | 0 |  |  |
|  | May 2022 | 100 | 100 | 100 | 85 | 0 | 7 | 0 | 0 | 0 | 0 | 0 | 0 | 0 | 28 | 0 | 10 |  |  |
|  | Sept. 2022 | 50 | 66 | 50 | 33 | 0 | 33 | 0 | 0 | 0 | 0 | 0 | 0 | 0 | 0 | 0 | 0 |  |  |

R= rural pools, U= urban pools
