# Supplementary material for: Hazard potential of Swiss Ixodes ricinus ticks: Virome composition and presence of selected bacterial and protozoan pathogens
Source: PLoS One. 2023 Nov 13;18(11):e0290942. doi: 10.1371/journal.pone.0290942 (PMC10642849; doi:10.1371/journal.pone.0290942)
Supplement: S3 Table — (DOCX) [file pone.0290942.s003.docx]

**S3 Table. Pool positivity (%) for non-viral pathogens in questing ticks of different gender/development stage.**

| **Canton** | **Stage** | **Rickettsia sp.** | | **Ehrlichia sp.** | | **Borrelia sp.** | | **Neoehrlichia mikurensis** | | **Babesia sp.** | | **Francisella**  **tularensis** | |
| --- | --- | --- | --- | --- | --- | --- | --- | --- | --- | --- | --- | --- | --- |
|  |  | **R** | **U** | **R** | **U** | **R** | **U** | **R** | **U** | **R** | **U** | **R** | **U** |
| **SO** | Female | 80.8 | - | 11.5 | - | 0 | 0 | 0 | 0 | 0 | 0 | 0 | 0 |
|  | Male | 69.2 | - | 65.4 | - | 3.8 | 0 | 0 | 0 | 0 | 0 | 0 | 0 |
|  | Nymph | 93.3 | 100 | 66.7 | 100 | 0 | 14.3 | 0 | 0 | 0 | 0 | 0 | 0 |
| **BE** | Female | 80 | 100 | 0 | 100 | 0 | 0 | 0 | 0 | 0 | 0 | 0 | 0 |
|  | Male | 100 | 75 | 0 | 75 | 0 | 0 | 0 | 25 | 0 | 0 | 0 | 0 |
|  | Nymph | 100 | 50 | 66.7 | 25 | 0 | 25 | 0 | 0 | 0 | 0 | 0 | 25 |
| **GE** | Female | 0 | - | 0 | 0 | 0 | 0 | 0 | 0 | 0 | - | 0 | 0 |
|  | Male | 0 | - | 0 | 0 | 0 | 0 | 0 | 0 | 0 | - | 0 | 0 |
|  | Nymph | 25 | 33.3 | 25 | 33.3 | 25 | 33.3 | 25 | 33.3 | 0 | 0 | 0 | 0 |
|  | Mixed adult^a^ | - | 100 | - | - | - | - | - | - | - | 10 | - | - |
| **VS** | Female | 0 | 50 | 0 | 0 | 0 | - | 0 | 0 | 0 | 0 | 0 | 0 |
|  | Male | 0 | 50 | 33.3 | 0 | 0 | - | 0 | 0 | 0 | 0 | 0 | 0 |
|  | Nymph | 66.7 | 100 | 66.7 | 0 | 33.3 | - | 33.3 | 0 | 0 | 0 | 0 | 0 |
|  | Mixed^b^ | - | - | - | - | - | 12.5 | - | - | - | - | - | 0 |
| **TI** | Female | 100 | 100 | 50 | 0 | 0 | 0 | 0 | 0 | 0 | 0 | 0 | 0 |
|  | Male | 100 | 100 | 0 | 0 | 0 | 0 | 0 | 0 | 0 | 0 | 0 | 0 |
|  | Nymph | 100 | 87.5 | 60 | 28.6 | 0 | 0 | 0 | 0 | 0 | 0 | 0 | 0 |
| **GR** | Female | 42.9 | 0 | 42.9 | 66.7 | 0 | 20 | 0 | 0 | 0 | 0 | 0 | 0 |
|  | Male | 12.5 | 16.7 | 25 | 50 | 0 | 33.3 | 0 | 0 | 0 | 0 | 0 | 0 |
|  | Nymph | 75 | 100 | 83.3 | 81.8 | 0 | 0 | 20 | 0 | 0 | 0 | 0 | 0 |
| **JU** | Female | 62.5 | 100 | 12.5 | 50 | 0 | 50 | 0 | 0 | 0 | 0 | 0 | 0 |
|  | Male | 35.3 | 50 | 11.8 | 0 | 0 | 0 | 0 | 0 | 0 | 0 | 0 | 0 |
|  | Nymph | 78.6 | 75 | 0 | 57.1 | 0 | 0 | 0 | 25 | 0 | 0 | 0 | 0 |
| **SG** | Female | 50 | 50 | 50 | 25 | 0 | 25 | 0 | 0 | 0 | 0 | 0 | 0 |
|  | Male | 33.3 | 100 | 22.2 | 0 | 0 | 0 | 0 | 0 | 0 | 0 | 0 | 0 |
|  | Nymph | 100 | 100 | 62.5 | 0 | 12.5 | 0 | 0 | 0 | 0 | 0 | 0 | 0 |
| **SH** | Female | 66.7 | 100 | 33.3 | 42.9 | 0 | 0 | 0 | 0 | 0 | 0 | 0 | 0 |
|  | Male | 100 | 100 | 50 | 40 | 0 | 0 | 0 | 0 | 0 | 0 | 0 | 0 |
|  | Nymph | 100 | 100 | 66.7 | 35.7 | 0 | 6.5 | 0 | 0 | 0 | 0 | 0 | 0 |
| **ZH** | Female | 100 | 100 | 66.7 | 50 | 0 | 0 | 0 | 0 | 0 | 0 | 0 | 0 |
|  | Male | 100 | 100 | 100 | 40 | 0 | 0 | 0 | 0 | 0 | 0 | 0 | 0 |
|  | Nymph | 100 | 100 | 100 | 80 | 20 | 13.3 | 20 | 0 | 0 | 0 | 0 | 0 |

^a^Mixed adult= females and males; ^b^Mixed= adults and nymphs (pooled together due to low number), R= rural pools, U= urban pools
