# Supplementary material for: Hazard potential of Swiss Ixodes ricinus ticks: Virome composition and presence of selected bacterial and protozoan pathogens
Source: PLoS One. 2023 Nov 13;18(11):e0290942. doi: 10.1371/journal.pone.0290942 (PMC10642849; doi:10.1371/journal.pone.0290942)
Supplement: S4 Table — (DOCX) [file pone.0290942.s004.docx]

**S4 Table. Viral and bacterial burden shown as p value in the tick pools according to different variables.**

| **Criteria** | ***Rickettsia*** | ***Ehrlichia*** | ***Borrelia*** | ***N.mikurensis*** | ***Babesia*** | ***F.tularensis*** | **TBEV** | **ALSV** |
| --- | --- | --- | --- | --- | --- | --- | --- | --- |
| **Urban/rural** | 0.01* | >0.05 | 0.01* | >0.05 | # | # | >0.05 | >0.05 |
| **SO** | >0.05 | >0.05 | >0.05 | >0.05 | # | # | >0.05 | >0.05 |
| **BE** | - | - | - | - | # | # | - | - |
| **GE** | 0.01* | >0.05 | 0.001** | >0.05 | # | # | >0.05 | >0.05 |
| **VS** | 0.001** | >0.05 | 0.01* | >0.05 | # | # | >0.05 | >0.05 |
| **TI** | >0.05 | >0.05 | >0.05 | >0.05 | # | # | >0.05 | >0.05 |
| **GR** | <0.001*** | 0.01* | >0.05 | >0.05 | # | # | >0.05 | <0.001*** |
| **JU** | <0.001*** | 0.01* | >0.05 | >0.05 | # | # | >0.05 | >0.05 |
| **SG** | 0.01* | >0.05 | >0.05 | >0.05 | # | # | <0.001*** | >0.05 |
| **SH** | >0.05 | >0.05 | >0.05 | >0.05 | # | # | >0.05 | <0.001*** |
| **ZH** | >0.05 | 0.01* | >0.05 | >0.05 | # | # | 0.001** | >0.05 |
| **Month** | 0.001** | >0.05 | >0.05 | >0.05 | # | # | >0.05 | >0.05 |
| **Year** | >0.05 | <0.001*** | >0.05 | 0.01* | # | # | 0.01* | 0.001** |
| **Male** | >0.05 | >0.05 | >0.05 | >0.05 | # | # | >0.05 | >0.05 |
| **Female** | 0.001** | >0.05 | >0.05 | >0.05 | # | # | >0.05 | >0.05 |
| **Adults** | 0.01* | >0.05 | >0.05 | >0.05 | # | # | >0.05 | >0.05 |
| **Nymphs** | <0.001*** | <0.001*** | >0.05 | >0.05 | # | # | >0.05 | 0.001** |

#*Babesia sp.* and *Francisella tularensis* not enough data
