# Supplementary material for: Hazard potential of Swiss Ixodes ricinus ticks: Virome composition and presence of selected bacterial and protozoan pathogens
Source: PLoS One. 2023 Nov 13;18(11):e0290942. doi: 10.1371/journal.pone.0290942 (PMC10642849; doi:10.1371/journal.pone.0290942)
Supplement: S5 Table — (DOCX) [file pone.0290942.s005.docx]

**S5 Table. Pool positivity (%) for TBEV and ALSV of the different gender/development stages of questing ticks.**

| **Canton** | **Stage** | **TBEV** | | **ALSV** | |
| --- | --- | --- | --- | --- | --- |
|  |  | **R** | **U** | **R** | **U** |
| **SO** | Female | 0 | 0 | 0 | 0 |
|  | Male | 0 | 0 | 0 | 0 |
|  | Nymph | 0 | 0 | 0 | 0 |
| **BE** | Female | 0 | 0 | 0 | 0 |
|  | Male | 0 | 0 | 0 | 0 |
|  | Nymph | 0 | 0 | 0 | 0 |
| **GE** | Female | 0 | 0 | 0 | 0 |
|  | Male | 0 | 0 | 0 | 0 |
|  | Nymph | 0 | 0 | 0 | 0 |
| **VS** | Female | 0 | 0 | 0 | 0 |
|  | Male | 0 | 0 | 0 | 0 |
|  | Nymph | 0 | 0 | 0 | 0 |
| **TI** | Female | 0 | 0 | 0 | 0 |
|  | Male | 0 | 0 | 0 | 0 |
|  | Nymph | 0 | 0 | 0 | 0 |
| **GR** | Female | 14.3 | 20 | 28.6 | 0 |
|  | Male | 0 | 16.7 | 25 | 0 |
|  | Nymph | 9.1 | 8.3 | 50 | 18.2 |
| **JU** | Female | 0 | 0 | 0 | 0 |
|  | Male | 0 | 0 | 0 | 0 |
|  | Nymph | 0 | 0 | 0 | 0 |
| **SG** | Female | 25 | 0 | 0 | 0 |
|  | Male | 33.3 | 0 | 0 | 0 |
|  | Nymph | 37.5 | 0 | 0 | 0 |
| **SH** | Female | 0 | 14.3 | 0 | 14.3 |
|  | Male | 0 | 0 | 0 | 16.7 |
|  | Nymph | 0 | 0 | 66.7 | 41.9 |
|  | Mixed adult^a^ | - | - | - | 25 |
| **ZH** | Female | 0 | 0 | 0 | 0 |
|  | Male | 0 | 20 | 0 | 0 |
|  | Nymph | 0 | 26.7 | 0 | 6.7 |
| **Total** | **Female** | **2.6** | **1.7** | **1.7** | **0.9** |
|  | **Male** | **2.4** | **1.6** | **1.6** | **0.8** |
|  | **Nymphs** | **2.4** | **3.0** | **6.1** | **9.8** |
|  | **Mixed adult^a^** | **0** | **0** | **0** | **4.5** |

^a^Mixed adult= females and males (pooled together due to low number); R= rural pools, U= urban pools
